# Traffic Flow Detection Using Camera Images and Machine Learning Methods in ITS for Noise Map and Action Plan Optimization

**DOI:** 10.3390/s22051929

**Published:** 2022-03-01

**Authors:** Luca Fredianelli, Stefano Carpita, Marco Bernardini, Lara Ginevra Del Pizzo, Fabio Brocchi, Francesco Bianco, Gaetano Licitra

**Affiliations:** 1 Physics Department, University of Pisa, Largo Bruno Pontecorvo 3, 56127 Pisa, Italy; 2 Institute of Chemical and Physical Processes of National Research Council, Via G. Moruzzi 1, 56124 Pisa, Italy; 3 iPOOL S.r.l., Via Cocchi 7, 56121 Pisa, Italy; stefano.carpita@i-pool.it (S.C.); lara.delpizzo@i-pool.it (L.G.D.P.); fabio.brocchi@i-pool.it (F.B.); francesco.bianco@i-pool.it (F.B.); 4 Institute of Marine Engineering of National Research Council, Via di Vallerano 139, 00128 Rome, Italy; marcobernardini.s@gmail.com; 5 Environmental Protection Agency of Tuscany Region, Via Vittorio Veneto 27, 56127 Pisa, Italy

**Keywords:** intelligent transportation systems, sound mitigation, noise maps, traffic measurements, machine learning, YOLO, vehicle detection, noise exposure, annoyance, G_den_

## Abstract

Noise maps and action plans represent the main tools in the fight against citizens’ exposure to noise, especially that produced by road traffic. The present and the future in smart traffic control is represented by Intelligent Transportation Systems (ITS), which however have not yet been sufficiently studied as possible noise-mitigation tools. However, ITS dedicated to traffic control rely on models and input data that are like those required for road traffic noise mapping. The present work developed an instrumentation based on low-cost cameras and a vehicle recognition and counting methodology using modern machine learning techniques, compliant with the requirements of the CNOSSOS-EU noise assessment model. The instrumentation and methodology could be integrated with existing ITS for traffic control in order to design an integrated method, which could also provide updated data over time for noise maps and action plans. The test was carried out as a follow up of the L.I.S.T. Port project, where an ITS was installed for road traffic management in the Italian port city of Piombino. The acoustic efficacy of the installation is evaluated by looking at the difference in the acoustic impact on the population before and after the ITS installation by means of the distribution of noise exposure, the evaluation of G_den_ and G_night_, and the calculation of the number of highly annoyed and sleep-disturbed citizens. Finally, it is shown how the ITS system represents a valid solution to be integrated with targeted and more specific sound mitigation, such as the laying of low-emission asphalts.

## 1. Introduction

The prevention of citizens’ noise exposure is fundamental in modern society in order to avoid the onset of health effects such as sleep disorders [1,2], learning impairment [3], diastolic blood pressure and hypertension [4,5], ischemic heart disease [6], and annoyance [7,8]. Road traffic is the most impacting source, with 82 million of Europeans affected by long-term day–evening–night (L_den_) traffic noise levels of at least 55 dB (A) [9]. The common guideline to prevent noise was outlined in 2002 when the European community issued the Environmental Noise Directive (END) [10]. In this document, acoustic mappings were prescribed in order to estimate the noise emitted in certain areas by the main noise sources. Acoustic mappings represent the first step toward the calculation of the number of citizens exposed to certain noise levels and, consequently, which mitigation actions should be planned, every 4 years, when noise exposures are close to health disturbances thresholds. In the noise mapping phase, models can calculate the propagation of noise in the environment only if appropriate input data are inserted. For road traffic [11,12,13,14], usual input data are represented by traffic flow and average speed for the different vehicles categories according to the CNOSSOS-EU model [15], as well as the type of road pavement.

In this light, many studies were devoted to the investigation of the parameters affecting road traffic noise. Besides engine noise, tire/road interaction—also known as tire/road noise—is the main generation mechanism acting in the speed range cars usually have in urban and suburban contexts [16]. Tire/road noise depends on both the tire model [17] and the type of road surface [18,19], since the effect of its properties such as road texture [20,21,22] and its job mix formula [23,24] can be considered when optimizing pavements from an acoustic point of view. Low-noise pavements represent an effective tool in road traffic noise mitigation, not only because they reduce noise acting on the sound power of the source, but also because, compared to other solutions—such as ordinary noise barriers—they yield a smaller impact on the surrounding environment and on the citizens. Usually, the mitigation of noise through new pavements occurs where barriers cannot be placed or are not accepted, but they can also be used in combination in order to maximize the insertion loss. In any case, the cost of the shares depends on their extent.

A further solution for noise reduction could be represented by Intelligent Transportation Systems (ITS), which represent the current and future perspectives in the field of road transportation. However, to the best of the authors’ knowledge, their potential as noise mitigation has been studied only marginally in the scientific literature by a couple of studies [25,26], not yet receiving the attention they deserve.

A number of different systems fall within the ITS category, all aiming to promote transport safety, mobility, and environmental sustainability. This is done by integrating communication and information technology applications into the management and operation of the transport system, in all its aspects. The objective of the ITS is to connect the various vehicles, the road infrastructure, the mobile devices of the passengers or specific instruments installed along the roads in order to warn drivers in real time about potential dangers or road conditions, guiding them towards different choices for the best route.

The usefulness of ITS has been reported in various sectors, but the best results are obtained in crash prevention, safety for connected vehicles, and driver assistance in automated vehicles [27]. Left turn assist, traffic control violation warning, and stop sign gap assist are among the in-road sensors that have helped reducing collisions with pedestrians [28]. The intra-vehicular sensing platforms—including vehicle-to-infrastructure (V2I), vehicle-to-vehicle (V2V), and vehicle-to-pedestrian (V2P) applications—have demonstrated success in detecting potential conflicts, warning drivers of crash potential and then reducing the risk of fatal crashes [29,30,31]. Data collected by sensors during V2V and V2I is then provided to transportation management systems for further processing and analysis and this process should be ensured by the highest quality sensor as the success of the ITS itself depends on how much the platform used to access, collect, and process accurate data from the environment is fast and easy to access [32,33]. The technology allowing vehicles to link to a wireless router to enable inter-vehicular communication firstly was the vehicular ad hoc network (VANET), which was later extended to the internet of vehicles (IoV) for interacting with ITS [34]. In fact, IoV is an intelligent communication link via mobile internet between vehicles and public networks that includes vehicular networking and vehicular intelligence for V2V and V2I communications [35].

For the purpose of the present work, the most important aspect is traffic monitoring and vehicle detection in ITS. Inductive loop detectors, radar detectors, and laser detectors are the most common sensors used to detect vehicles [36], but their main drawbacks can be found in maintenance cost and environmental issues [37]. Video cameras are cheaper and more flexible than these traditional sensors and the increasing numbers of closed-circuit television (CCTV cameras) have boosted image-based vehicle detection as a technique for large-scale traffic information data collection.

Many years have passed since video-based vehicle detection was applied to ITS [38] to provide information assisting vehicle counting, vehicle speed measurement, identification of traffic accidents, and traffic flow prediction. In summary, the ITS dedicated to traffic management uses sensors for the acquisition of traffic data that act as inputs to traffic models updated in real time, with subsequent warnings to drivers through various systems to avoid certain events or to be guided towards more effective routes. Seen from this perspective, ITS systems and acoustic mapping present similar inputs, although they are different and have different functionalities. It would be interesting for the two activities to be integrated; for example, by ensuring that the ITS cameras could collect traffic data also according to the action plan and the acoustic maps, thus acquiring traffic data according to the specifications provided by the END [10].

Video analysis methods for vehicle detection and traffic monitoring should overcome various challenges which are faced with many different methods [37,38]. The main difficulties arise from the dynamic observation conditions related to illumination changes during daytime and nighttime, different weather conditions, shadows produced by vehicles or objects, and vehicle occlusions. Classical machine learning appearance-based methods are based on the recognition of specific local features using descriptors such as the histogram of gradient (HOG) [39,40], Haar-like features [41], Gabor features [42], speed-up robust features (SURF) [43], and scale-invariant features transform (SIFT) [44]. The feature recognition is usually combined with the use of classifiers such as support vector machines (SVM), decision trees classifier, artificial neural networks based on multilayer perceptron architecture, and ensemble methods such as Adaboost or Random Forest [40,45]. Other classical motion-based methods include background modeling methods, using for example Gaussian mixture models, background subtraction, or optical flow [46,47]. The main drawback of these methods is that they are feature dependent: rapidly changing observation conditions could decrease detection performances and should be studied thoroughly for each case. These algorithms often require hand-crafted parameter adjusting and optimization by human expertise to best represent features of the target objects [37].

Nowadays, thanks to the development of deep neural networks (DNN) and the continuous improvement of GPU computation performances, it is possible to approach the problem of vehicle detection differently. Deep learning methods are able to extract features directly from original images or video, without the need for detailed analysis of different conditions and circumstances. The downside of these methods is the requirement of large amount of labeled data to proceed to the model training in a supervised manner. The data should be collected and prepared for the various environmental conditions in order to achieve good detection and classification performances. Anyhow, the availability of labeled datasets for vehicle detection is increasing thanks to the growth of the research on autonomous driving vehicles and machine learning methods [48,49,50,51].

Among several deep learning methods, the YOLO (you only look once) object detection model family [52,53,54,55,56] introduced a new architectural approach that leads to a significant improvement—especially in computation speed—and an easier implementation of real-time analysis systems [57,58,59]. 

In this work, a video measurement system (VMS) for vehicle detection and classification based on a tracking-by-detection approach and the YOLOv2 [53] model is presented. The VMS consists of a low-cost video recording system (VRS) and a video analysis system (VAS). The VMS is used to perform roadside measurements, mainly related to environmental acoustics, with applications to noise mappings and statistical pass-by (SPB) or controlled pass-by (CPB) measurements [60,61]. In particular, the system is designed to detect and classify vehicles according to the categories defined in the CNOSSOS-EU model [15], required by the END [10] for road infrastructures.

The developed video measurement system has been tested and used, together with other standard sensors, to perform traffic measurements in Piombino (Italy) as a follow-up of its acoustic mapping performed inside the INTERREG Maritime Programme Italy–France 2014–2020 [62] L.I.S.T. PORT [63].

The input data for the noise model were acquired with specific short- and long-term noise and traffic measurements performed with sound level meters and the specific designed VMS. The measurements and maps have been carried out in two different periods: the summer 2019 peak period and the summer 2021 peak period. During this 2-year period, the project installed an info-mobility ITS that automatically provided the most appropriate directions to drivers arriving and leaving the port area.

The traffic flows before and after the installation of the system are compared and then, by means of noise maps, the consequent changes in the acoustic footprints in the area are estimated. The overall effectiveness of the mitigation action is also evaluated in terms of citizens’ noise exposure by means of the G_den_ and G_night_ indicators [64,65], and with the total number of citizens that are highly annoyed and sleep-disturbed.

## 2. Video Measurement System

The acoustic mapping of road infrastructure requires the measurement or estimation of traffic flow and speed, for each vehicle category defined in the CNOSSOS-EU model [15] (i.e., cars, medium–heavy trucks, heavy trucks, motorcycles, and mopeds). Several sensors are available to measure traffic flow and speed, such as magnetic sensors, infrared sensors, photoelectric sensors, Doppler and radar sensors, inductive loops, and video camera systems.

**Figure 1:**
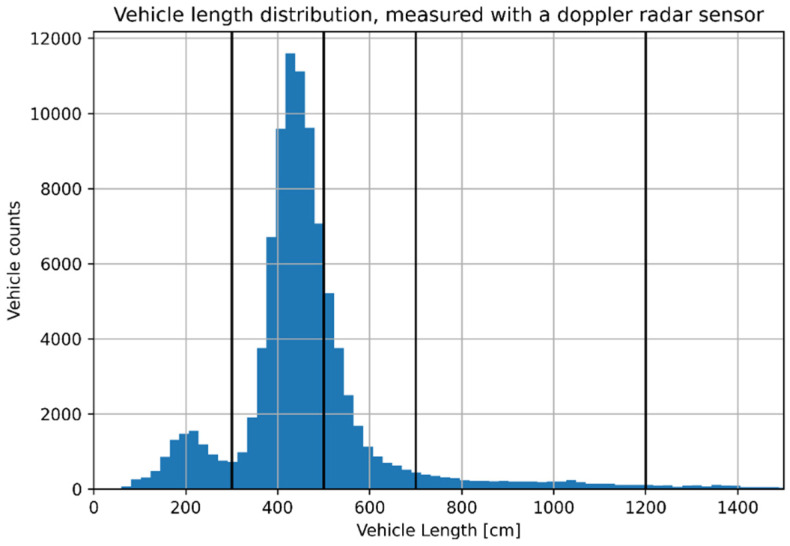
Vehicle length distribution measured using a radar Doppler sensor used in Piombino.

The measurement system for a single vehicle passing by should perform two different tasks: vehicle classification and vehicle speed measurement. While speed measurement could be performed with the various sensors with similar results, vehicle classification is more cumbersome. Standard measurement systems usually achieve the task of classification by measuring the length of the vehicle: for example, a radar sensor uses the Doppler effect by measuring the change of frequency of low energy microwave radiation reflected by vehicles, estimating both vehicle speed and length. The distribution of vehicle length measured with a radar Doppler sensor for a single measurement performed in Piombino is shown in Figure 1. The two peaks correspond to the average length for motorcycles and cars. The vertical lines show an example of vehicle classification performed by splitting the continuous length distribution in categories with typical vehicle dimensions.

However, classification of vehicles based on their length could easily lead to misclassification of vehicles with similar length and misclassification errors due to the choice of the length range for each category. While misclassification errors do not affect greatly traffic circulation monitoring, from the point of view of noise emission an improvement in classification performance could be useful to distinguish, e.g., mopeds from motorcycles or medium trucks from long cars. The problem of vehicle classification and counting could be faced by using video analysis systems and modern machine learning methods.

Hashemi et al. [66] presented a literature survey of vehicle detection and classification (VDC) methods based on artificial neural networks reported from 2012 to 2021. The paper also introduces a framework to compare different approaches, based on the definition of nine characteristics of VDC systems. The video measurement system (VMS) presented in this paper consists of a video recording system (VRS) and a video analysis system (VAS). In the following, the designed VMS is described using the dimensions of the comparison framework in [66].

### 2.1. Application

The VMS is designed for traffic monitoring related the environmental noise field, with applications to noise mappings, roadside SPB, and CPB measurements [56,57], in addition to traffic monitoring. The system has been designed to adhere to the following requirements:

- The VRS should be easily installable at roadside, using a movable experimental apparatus also including the noise measurement equipment.
- The VAS should permit the vehicles classification using the category defined for the CNOSSOS-EU model [15].
- The measurement system should be based on low-cost hardware to easily produce multiple monitoring stations. The hardware cost should be much less than the system development cost.
- The system should perform measurements of the vehicle speed.
- The video analysis system could process the video recordings offline in order to maintain a simple measurement system, with a power autonomy of at least one week. Real-time processing performances should be possible, in case of installation in fixed monitoring stations.

### 2.2. Input Source

The VMS is based on low-resolution video recordings (640 × 480 p). Low resolution and the chosen framing are sufficient to perform vehicle detection and classification and at the same time allow the device to comply with the ‘privacy by design’ principle, since the system is not able to perform license plate or facial recognition. The storage resources are therefore limited, and the data management consequently is easier.

### 2.3. Vehicle Type

The system permits the classification of the vehicles categories defined for the CNOSSOS-EU model [15]: light motor vehicles, medium heavy vehicles, heavy vehicles, and powered two wheelers (mopeds and motorcycles). 

The categories recognized by the VAS are more specifics in order to better differentiate between the visual features, and include car, SUV, open van, motorcycle, moped, truck, bus, van, and box van.

### 2.4. Scope/Domain

The data are acquired by movable roadside monitoring stations, used for traffic monitoring in the context of environmental acoustics applications.

### 2.5. Dynamicity

In terms of the appearance characteristics, the system could be considered static, because it is based on the tracking-by-detection approach, where vehicle detection and classification are performed on single images and static features.

### 2.6. Evaluation Method

The system has been evaluated using a test set extracted from an overall dataset consisting of a total of 14,400 labeled images—divided into 8000 images gathered in daylight conditions and 6400 at night—labeled by human operators for the object detection task. The metrics used for evaluation are the mean average precision and the log average missing rate.

### 2.7. Scale

The scale of the system in terms of operation domain, time complexity, and adaptability could be considered medium. The system is adaptable to different applications, respecting the design requirements. The system works well and has been tested for roads with at most two to three lanes, using a roadside lateral view.

### 2.8. Vehicle Detection Method and Vehicle Classification Method

The tasks of vehicle detection and classification are solved jointly by using a YOLOv2 object detection model [53], and a pretrained convolutional neural network.

#### 2.8.1. Video Recording System

**Figure 2:**
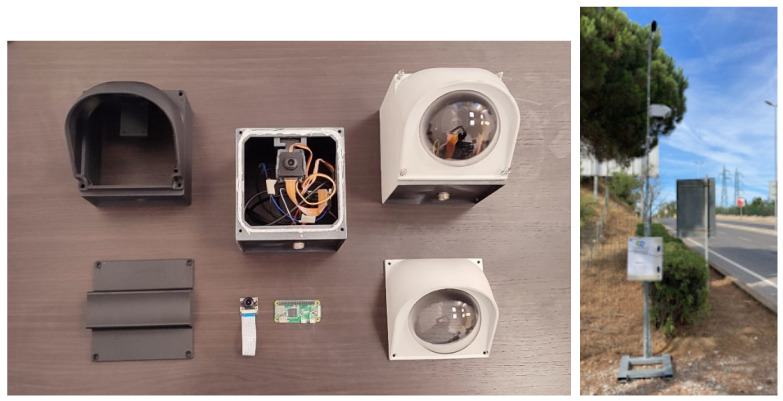
Video camera system using a single-board computer, an infrared wide-angle camera, and a 3D-printed casing and its application with sound level meter and video camera system at roadside in Piombino.

The video recording system (VRS) is based on a Raspberry Pi single-board computer, equipped with a wide-angle camera lens. The camera casing shown in Figure 2 has been designed on a CAD model and was manufactured using a 3D printer. The camera is controlled via Wi-Fi and a pan–tilt system is used to compose the framing.

The camera is mounted together with a sound level meter, at a height of about 3–3.5 m above the ground. The system is powered by batteries that allow the system to measure continuously for at least one week. For night measurements, the camera exposure parameters are set to increase its sensitivity. Furthermore, the camera is sensitive to infrared light, so that in cases of absence of artificial illumination IR illuminators could be used. The frame rate of the recordings is equal to 30 fps for daytime and 20 fps for nighttime. In order to easily install the measurement system in different urban areas, the camera system is mounted on the side of the roads as shown in Figure 2, the video traffic monitoring uses a lateral view, differently from more usual permanently installed video systems.

#### 2.8.2. Video Analysis System

The problem of vehicle detection and tracking using video analysis is a specific case of the general task of multiple object tracking (MOT), which plays an important role in computer vision and has applications in various fields [67,68]. The MOT task could be approached by adopting the so-called tracking-by-detection strategy. In this methodology, the objects are first detected in a single video frame and then are linked by using another tracking algorithm. The video analysis system (VAS) presented in this paper uses this approach, which could be divided in the following sub-tasks:

1. Detection—The vehicles in each video frame should be located. A single detection result could be a bounding box containing the object or an irregular shape obtained by the image segmentation;
2. Classification—Each vehicle detected in a single frame should be classified in well-defined categories;
3. Tracking—The unique identity of a single vehicle should be maintained frame by frame, in order to track it. For this task, the problem of vehicle superposition or hiding should be faced;
4. Distance measurement—The camera system should be calibrated in order to transform distances measured in pixel units to real-world units, allowing vehicle speed measurement.

**Figure 3:**
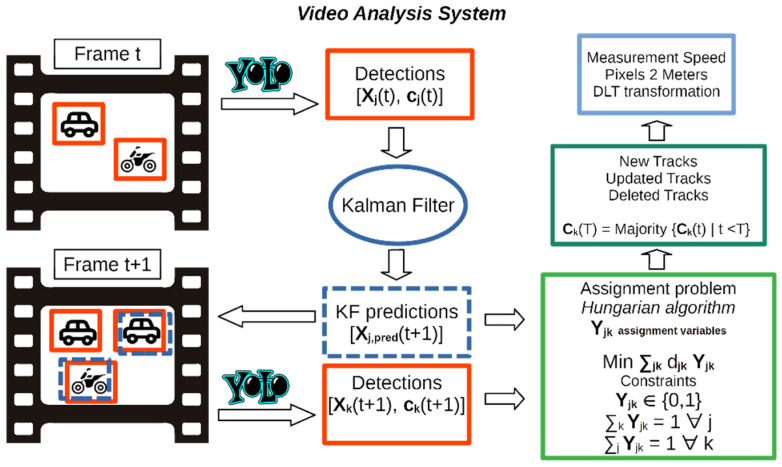
Video analysis system schematic drawing.

The VAS, schematized in Figure 3, solves the detection and classification tasks jointly using the YOLOv2 object detection model [53]. For each frame *t*, YOLOv2 predicts the bounding boxes containing the j detected vehicles, yielding their pixel positions $X_{j}\left(t\right)$ and the vehicles categories $c_{j}\left(t\right)$. The YOLO model (you only look once) presented by Redmon et al. in [52] was the first object detection model based on deep neural networks which reformulated the detection and classification task as a single regression problem. The network architecture consists of a single convolutional network which, from image pixels, directly predicts multiple bounding boxes and class probabilities at the same time. The new approach largely improved speed performances of previous detectors, allowing real time detections on fast GPUs. The model was updated to version YOLOv2 with the introduction of anchor boxes, pre-defined bounding boxes which allow better object location performances [53]. YOLOv3 introduced further modification, such as a feature pyramid network and a binary cross-entropy loss function, to improve the detection accuracy and the ability of detecting smaller objects [54]. Joseph Redmon—main author of YOLOv1, YOLOv2, YOLOv3—quit his research on YOLO detectors because of broader impact concerns, such as privacy and possible military applications, as anticipated in the conclusions of Redmon and Farhadi in 2018 [54]. Further development of the architecture has been presented in models YOLOv4 and YOLOv5 [55,56]. The use of the YOLOv2 model achieved good performance for the VAS, in the future also newer YOLO models will be tested and evaluated.

YOLO models, as with most machine learning methods based on artificial neural networks, learn to tackle its task in a supervised manner—i.e., by using as input labeled data. Achieving good performances using deep learning methods usually requires a large amount of labeled data and images for the task at hand. The principle of transfer learning is quite useful to reduce the amount of new labeled data needed to train the model. For the VAS, a pre-trained ResNet-50 convolutional neural network has been used to build the YOLOv2 architecture [69]. The pre-trained network is trained on more than a million images from the ImageNet database, and it could classify images into 1000 categories [48].

**Figure 4:**
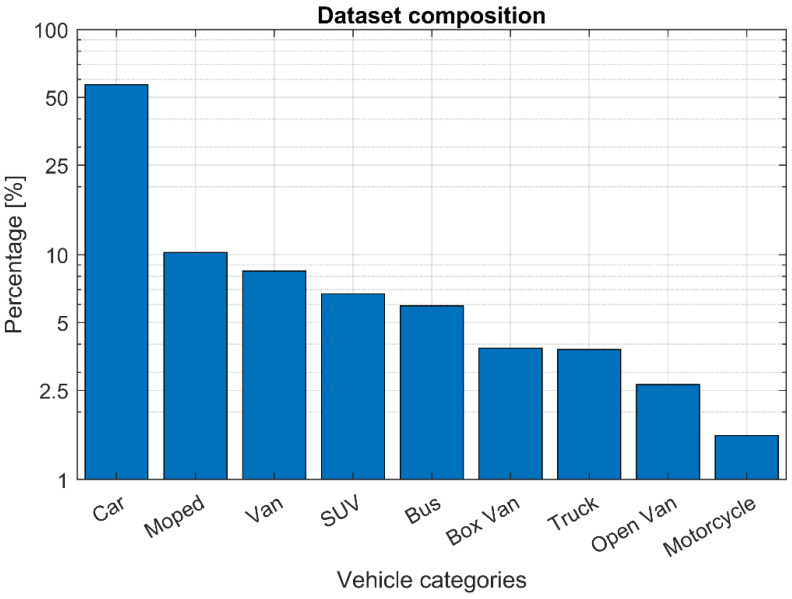
Dataset composition by vehicle categories. The dataset contains about 14,400 labeled images—8000 gathered in daylight conditions and 6400 at night—labeled by human operators for the object detection task.

In order to specialize the detection and classification abilities of the VAS to vehicle recognition the model has been retrained using a dataset of images extracted from videos recorded in 20 different positions during several traffic measurement campaigns. The overall dataset consists of a total of 14,400 labeled images—divided into 8000 images gathered in daylight conditions and 6400 at night—labeled by human operators for the object detection task. The composition of the dataset by vehicle category is represented in Figure 4. The YOLOv2 model is retrained depending on the measurement conditions to analyze, by extracting a dataset from the main images collection, which is usually balanced for vehicle categories, using oversampling of minority classes.

In order to count the vehicles, it is necessary to maintain the identity of each detected vehicle from frame to frame. The VAS uses as tracking algorithm a Kalman filter combined with the so-called Hungarian algorithm [70]. The Kalman filter is used to predict the position of a vehicle in the next frames, using a simple constant speed motion model and the bounding boxes positions in previous frames, computed by the YOLOv2 detector.

**Figure 5:**
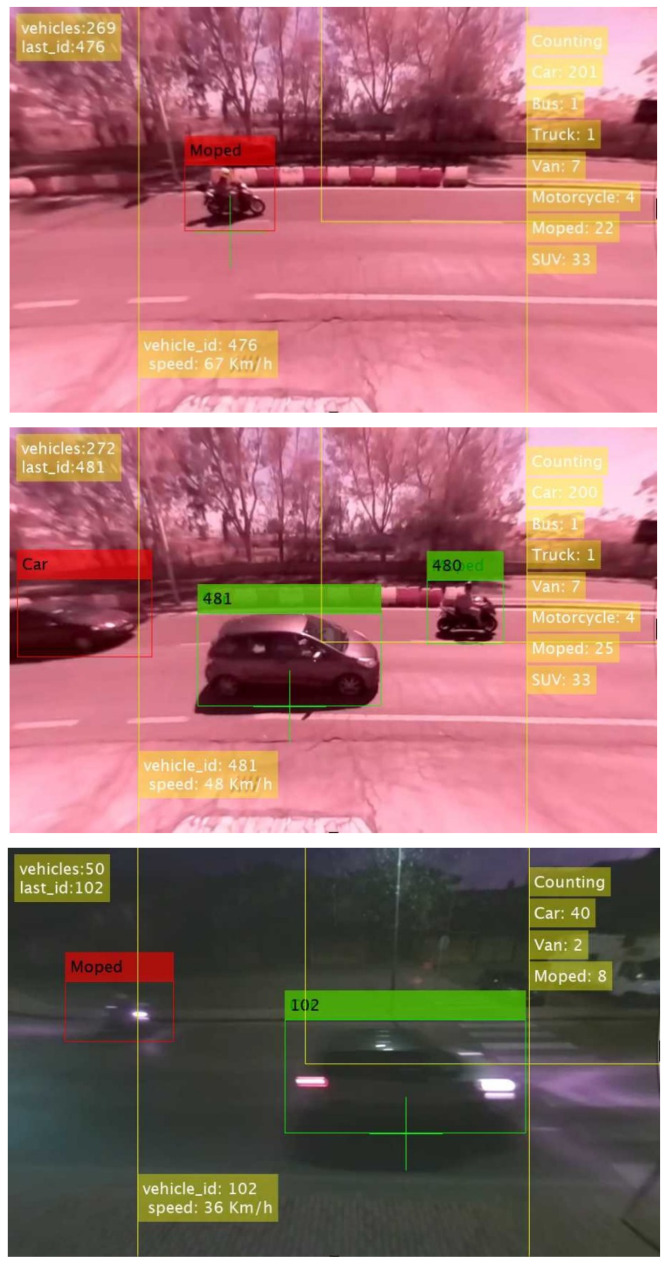
Examples of video processing via tracking-by-detection for daytime and nighttime.

The Hungarian algorithm associates the IDs of the vehicles detected in the previous frame to the new YOLOv2 detections in the current frame, by minimizing the pixel distances d_jk_ between the Kalman predicted positions and the new bounding boxes centroids, as shown in Figure 3. The linear programming assignment problem, solved via the Hungarian algorithm, can perform the vehicle tracking by establishing the association between detections in different frames and creating a unique track for each vehicle. The number of detections in subsequent frames could be different because of the appearance of new vehicles or their movement outside the video framing. Depending on the solutions of the assignment problem, the VAS could create a new track, update the existing tracks with new positions and classes, or delete some of the current tracks. For each vehicle, the category is assigned by considering the majority of the YOLOv2 predicted classes for a single track. The tracking algorithm is quite simple but is sufficient to handle basic occlusions of vehicles moving in opposite directions in most cases. The algorithm is also optimized by some ad-hoc strategies to reduce ids swapping errors. In Figure 5, an example of video processing is shown.

The speed measurement is performed by transforming the vehicles’ position coordinates expressed in pixel units to real-world units. The transformation is based on the pinhole camera model and on the estimation of a camera projection matrix by using a direct linear transformation (DLT) algorithm. In order to estimate the matrix for a single video, a grid of reference points is built on the frames. The real-world coordinates of the grid on the road plane are set by taking marks on the road, or by using vehicle dimensions as reference for the calibration. Furthermore, the distortion of the wide camera lens is corrected by a proper calibration, allowing a linear coordinates transformation.

**Figure 6:**
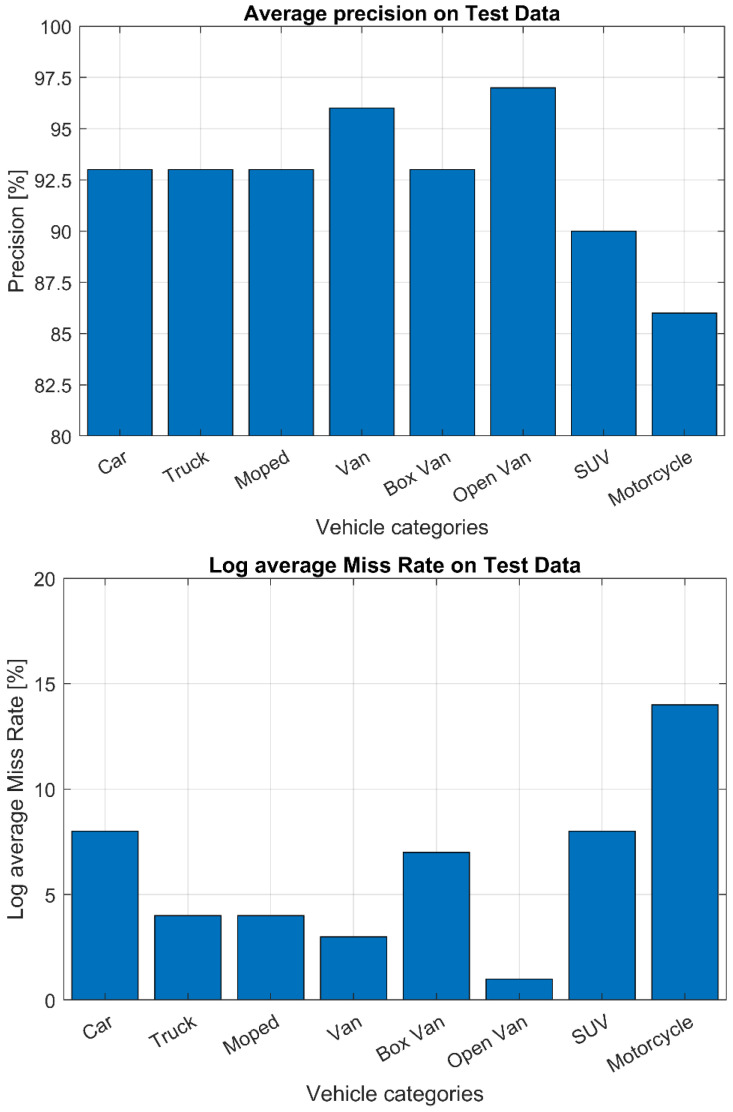
Detection average precision and log average miss rate of the trained Yolov2 model for different vehicle categories, evaluated on a test set.

In Figure 6, the detection average precision on a test set is reported for each vehicle category. The test set consists of 1500 labeled images extracted from the main collection. The precision is computed using an intersection over union threshold equal to 0.5. The mean average precision results equal to mAP = 92%.

The VAS tracking algorithm introduces some errors due to ids swapping, decreasing the detection precision depending on the measurement specific conditions, but on average the VAS precision is higher than 90%. In the future, a more extensive validation of the VAS measurement method will be presented, together with a comparison to other measurement techniques.

## 3. Real Case Test

### 3.1. Area under Study

**Figure 7:**
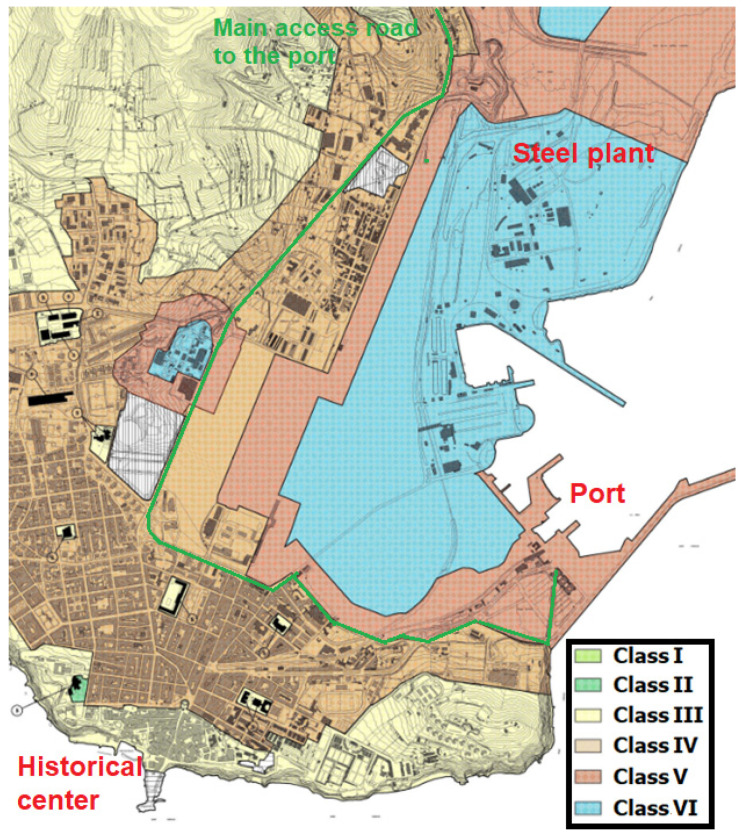
Acoustic territorial zoning of Piombino.

The study took place in Piombino, an Italian municipality of about 35,000 inhabitants in the province of Livorno (Tuscany), in front of the Island of Elba and at the northern side of the Tuscan Maremma. The city has always been an important port since the Etruscans, who left an ancient historical center, and, in modern times, the second-largest steel plant in Italy was also built within the city boundaries, with an area that covers almost 12,000,000 m² and 9 km of coastline. Given its strategic position, its port is still heavily used, both for industry and for tourism, with ferries from and to the Island of Elba, Olbia (Region of Sardinia), Bastia (Corsica, France), and other islands of the Tuscan Archipelago. Therefore, a large flow of seasonal tourist traffic crosses the city along its main route, Viale Unità d’Italia (SS398), in order to reach the boarding points at the port. Figure 7 reports the acoustic territorial zoning of Piombino, according to the Decree by the Prime Minister of Italy in 14 November 1997 [71], and the critical points for the study.

### 3.2. Collection of Preliminary Data

Gathering the geo-referenced cartographic documentation made available by the Municipality of Piombino and implementing them onto a GIS platform is the starting point for noise maps work. Among the most important features required are:

- Boundaries of the study area.
- Road network, retrieved from the website of the Municipality of Piombino [72], double checked with the dataset of the regional roads [73] in order to verify the geometries or to correct missing road sections. Each road section was then filled with the traffic flow information gathered with the methodology described in Section 3.
- Updated building planimetry of the area, with particular attention to their height, taken from both [72] and [73].
- Elevation points, in shapefile format of the area of interest, acquired from the online databases to build the digital 3D terrain model (DTM) for the sound propagation model.
- Ground absorption, retrieved by the land use (Corine Land Cover), obtained from [74].
- Census sections of the Municipality of Piombino and population data, available online at the Statistical National Institute [75]. Each inhabited building was then assigned a number of inhabitants proportional to its volume. The total number of citizens living in the studied area is 32,066.

### 3.3. Noise and Traffic Measaurements

**Figure 8:**
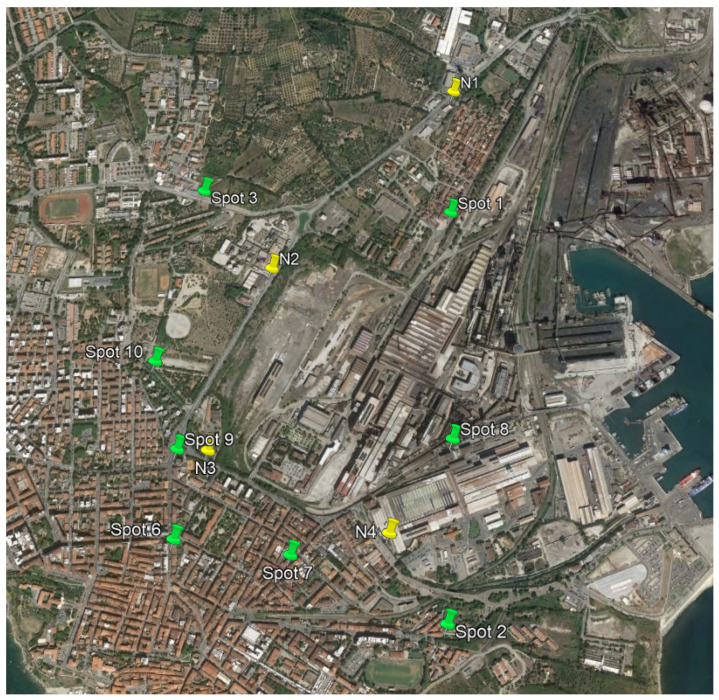
Aerial picture of Piombino with the positioning of long term (yellow) and short term (green) measurements. “Spot” measurements are the short-terms one, while the “N”s are the long-term.

The measurement campaign took place for 2 weeks over two sessions: ante-operam peak period (Summer 2019) and post-operam peak period (Summer 2021). In each session, four long-term and eight simultaneous short-term measurements were performed in the sites reported in Figure 8 which were used to validate the noise maps.

In each position, sound pressure level was acquired every 100 ms with class 1 sound level meter according to IEC 61672-1 [76], placed at 4 m above the ground level. The instrumentation was placed at the roadside using a source-oriented approach, since the aim of the work was characterizing the road noise source. A weather station was also installed for the entire duration of the surveys, in order to acquire rain, humidity, wind direction and speed, and air temperature. In the post-processing analysis, periods with rain or wind speed higher than 5 m/s were excluded. Moreover, unwanted events—such as animal or anthropic sounds—were manually removed by an operator analyzing the time history of sound pressure levels recorded.

Long-term measurements lasted for 7 days, while the short-term measurements lasted for at least one hour. The short-term measurement reports included the overall L_Aeq_; the statistical levels L_90_, L_50_, and L_10_; and the time history. The long-term ones included the same overall values per hour and the day, evening, and night level averaged over the entire measurement period, together with the plot of the hourly trend of the L_Aeq_.

Traffic data were acquired with the VRS—as described in Section 2—simultaneously with noise measurements.

### 3.4. Noise Mapping

The data acquired thanks to the methodology described in Section 2 represent the input data for the noise prediction model, implemented into a commercial noise simulation software. The noise model selected for this work is the CNOSSOS-EU: 2015 [15], compliant with Directive 996/2015/EU [77]. For road traffic noise prediction, the model requires traffic information for five different categories of vehicles (cars, medium–heavy trucks, heavy trucks, motorcycles, and mopeds).

The sound source considered in the present work is represented by the road network that affects the port waterfront and the roads nearby. The acoustic characterization of the latter was obtained by implementing average speeds and traffic flows acquired during the monitoring and by carrying out a calibration of the sources with the measured sound levels. The roads close to the waterfront were not monitored and were acoustically characterized following the guideline “Good Practice Guide Vol.2” of the European Commission WG-AEN Working Group [78]. These guidelines provide criteria for the assignment of traffic flows based on a categorization of roads. The validation process based on the results obtained showed that the model describes the acoustic climate of the investigated area with sufficient accuracy, as the differences found by comparing the simulated sound levels and the results of the measurements are contained, confirming the adherence of the calculation hypotheses to the investigated situation.

The simulations were performed considering 1 order of reflection, 500 m as the maximum search radius, 100 m as the largest distance of reflections from receiver, 50 m as the largest distance of reflections from source, a grid spacing equal to 10 m, and a height of 4 m.

The noise maps were reported for each of the END [10] indicators:

- L_d_—(6:00–20:00);
- L_e_—(20:00–22:00);
- L_n_—(22:00–06:00);
- L_den_—overall daily weighted.

### 3.5. ITS

The L.I.S.T. PORT has prescribed, for Piombino, the traffic monitoring in the main access and exit roads from the port, with the aim of defining a virtual model that can simulate new scenarios of the road network. This would imply reducing the vehicular load and therefore potentially reducing the noise impact of traffic.

**Figure 9:**
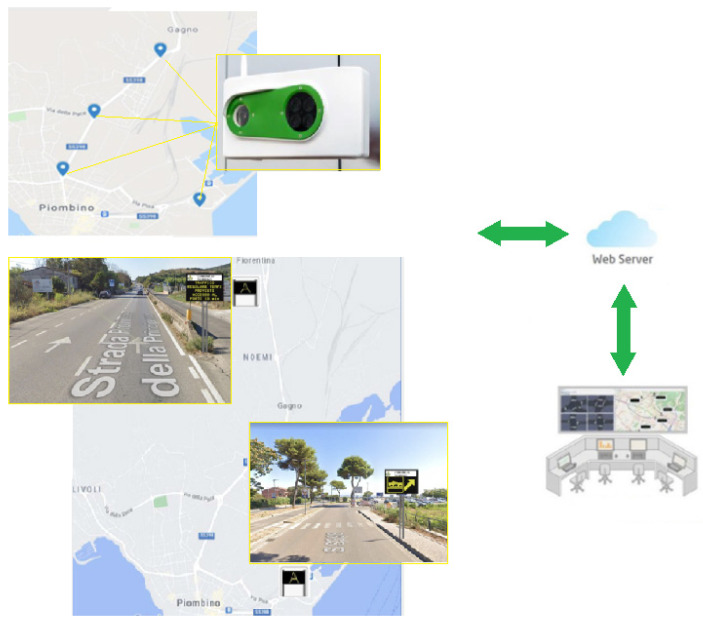
Representation and location of the ITS system implemented in Piombino.

The mitigation action implemented, and sketched in Figure 9, consists of a modular ITS system capable of monitoring and management of the different types of devices. The ITS system is composed by the following components:

- video camera systems for monitoring the characteristic parameters and the classification of traffic flow, consisting of four relevant positions on the road sections;
- variable-message signs and remote management system capable of providing information based on the traffic conditions detected by the supplied video camera system;
- processing unit for connection with cameras and variable-message signs;
- communication system with equipment for connectivity to the central system;
- signs and labels indicating a monitored/video surveillance area.

A system for acquiring information detected by road traffic monitoring stations sends messages to the citizen on variable-message signs to limit traffic and recommend alternative routes in case of traffic jams. Through a software platform for traffic and mobility management in the port city of Piombino, a list of tasks is possible: the representation of the road axis affected by heavy traffic, the collection of information from all systems, data processing with traffic status, detection of critical issues, and development ofscenarios to be implemented in certain conditions.

The server software performs two macro functions:

- The interface between server and field units (traffic monitoring stations, traffic light controllers, variable-message signs, underpasses, etc.);
- The interface between server and user workstations (client).

The messages displayed on the two variable-message signs are automatically loaded through different modes dependent on different parameters, such as the ferry schedule, the traffic situation identified within the specific scenario detected by the monitoring stations, the number of free parking slots, and estimated travel time. The messages can also be chosen among a series of default messages or can be additionally created by an operator. On the platform, simultaneous viewings of the variable-message signs are possible. Among other features, the platform allows the operator to visualize traffic data, real-time monitoring, and historical data and, moreover, provides analysis and processing related to mobility, with the aim of improving the knowledge about the characteristics of road traffic in Piombino and studying better solutions for mobility in port and urban area.

## 4. Results

**Figure 10:**
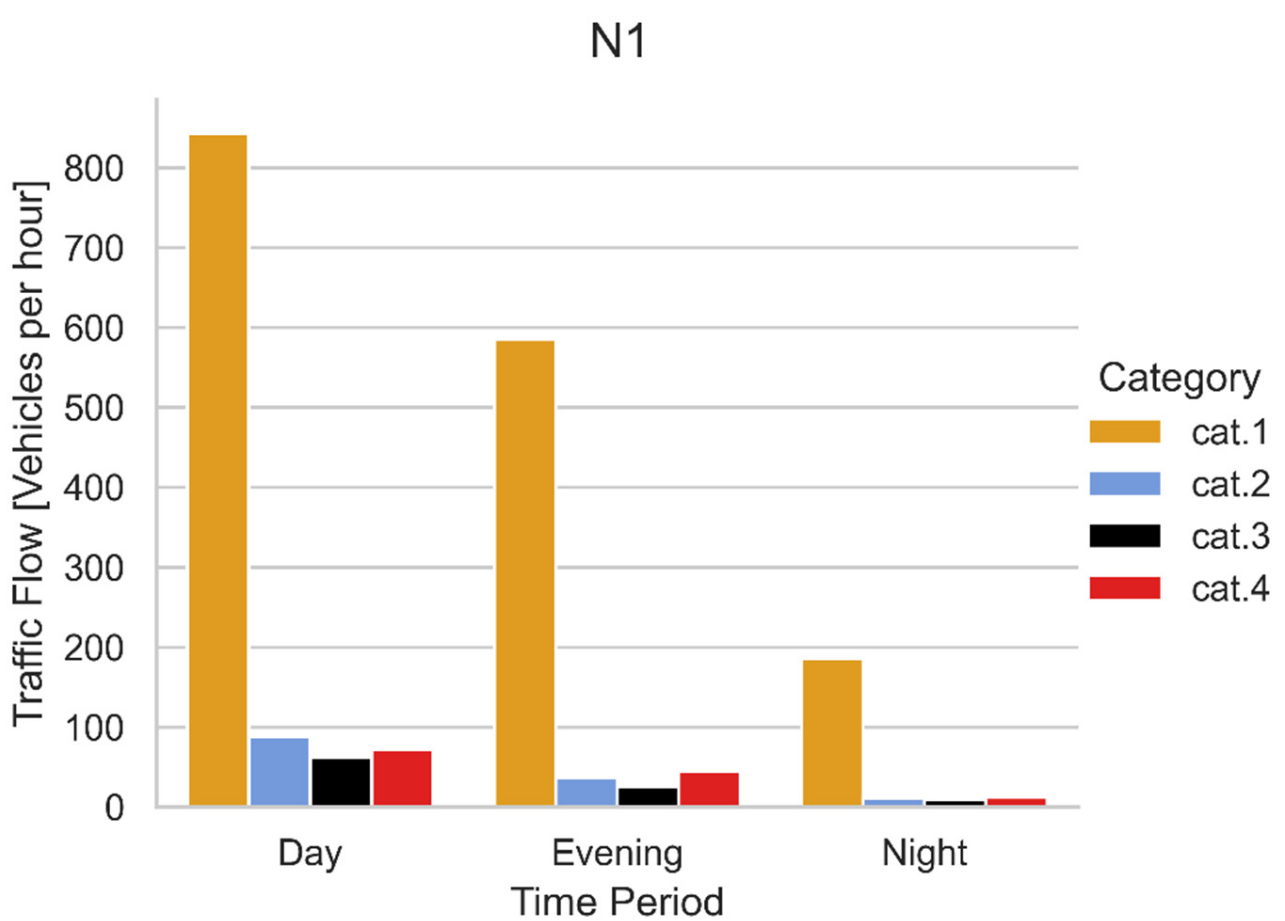
Traffic flow divided for categories of CNOSSOS-EU [15] and period of the day measured in N1 position in 2021.

**Figure 11:**
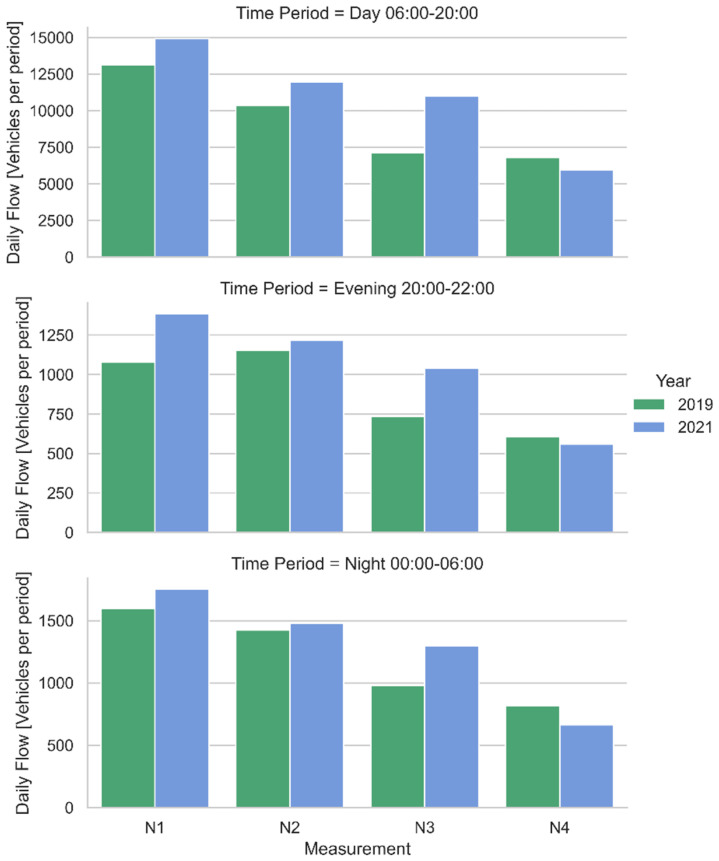
Overall traffic flow measured in positions N1–N4 for both years 2019 and 2021 divided into period of the day.

Figure 10 reports an example of traffic divided into the different CNOSSOS-EU [15] categories, obtained with the methodology describe in Section 3. Figure 11 shows the traffic flows measured at the points corresponding to the continuous noise measurements (N1–N4).

As it can be inferred from Figure 9, the number of vehicles circulating around Piombino in 2019 is far lower than in 2021. This could probably be due to a change in the holiday destinations considered by Italians, favoring national tourism after the COVID-19 pandemic and it is obviously not caused by the ITS, which can only redistribute the traffic among the different routes.

In order to evaluate the effectiveness of the ITS installation, the authors opted for a normalization of traffic flows to the same situation: in this way, all subsequent analyses results were comparable. The year 2019 was chosen as the reference period to normalize to, because it is just before the pandemic.

The normalization was therefore carried out by calculating, for the year 2021, the per-centage of vehicle flow for each vehicle category and period. This was possible thanks to the particular geography of Piombino, where only a single road access to the city is pre-sent. The traffic during 2021, normalized to 2019, was therefore obtained using data from 2019 to estimate the traffic entering the city, broken down in the road graph according to the percentages of 2021.

From now on, for the sake of clarity, the present article refers to the normalized 2021 scenario when it mentions the year 2021.

**Figure 12:**
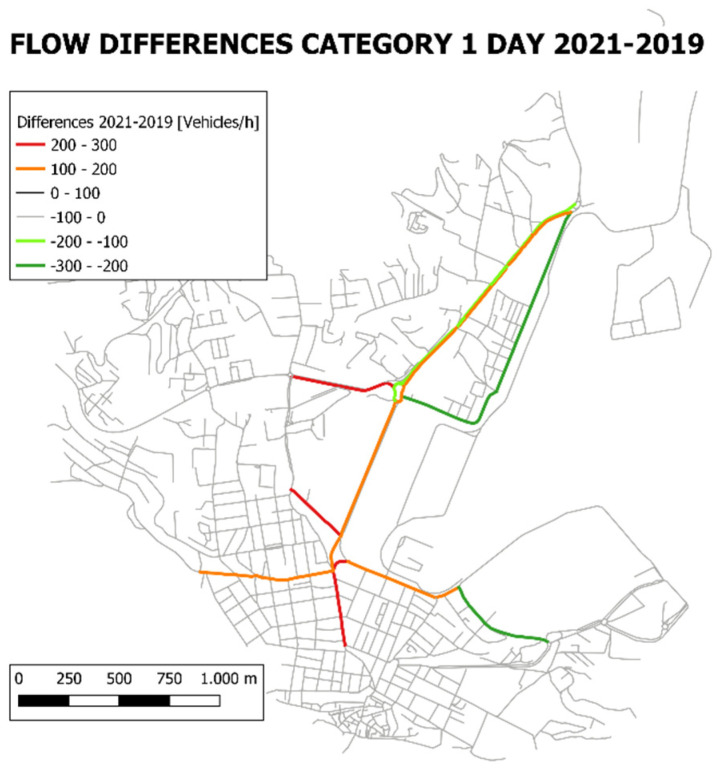
Road graph highlighting the differences in flows between 2021 and 2019 for vehicles category 1 in day period.

Figure 12 reports the road graph with highlighted the differences between the traffic flows of 2021 and 2019. Day period and category 1 is chosen as an example. 

**Figure 13:**
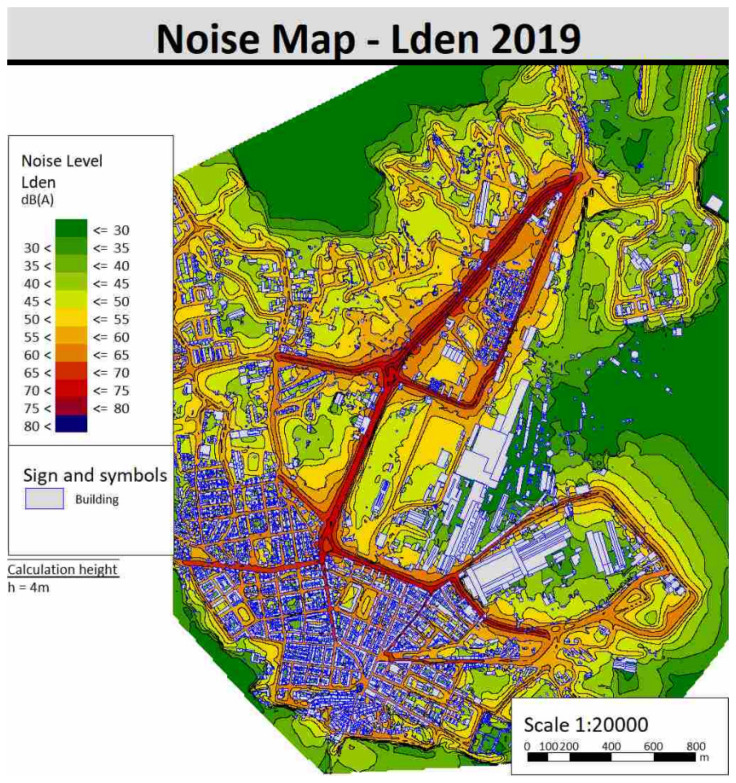
Noise maps of Piombino with L_den_ indicator for 2019.

**Figure 14:**
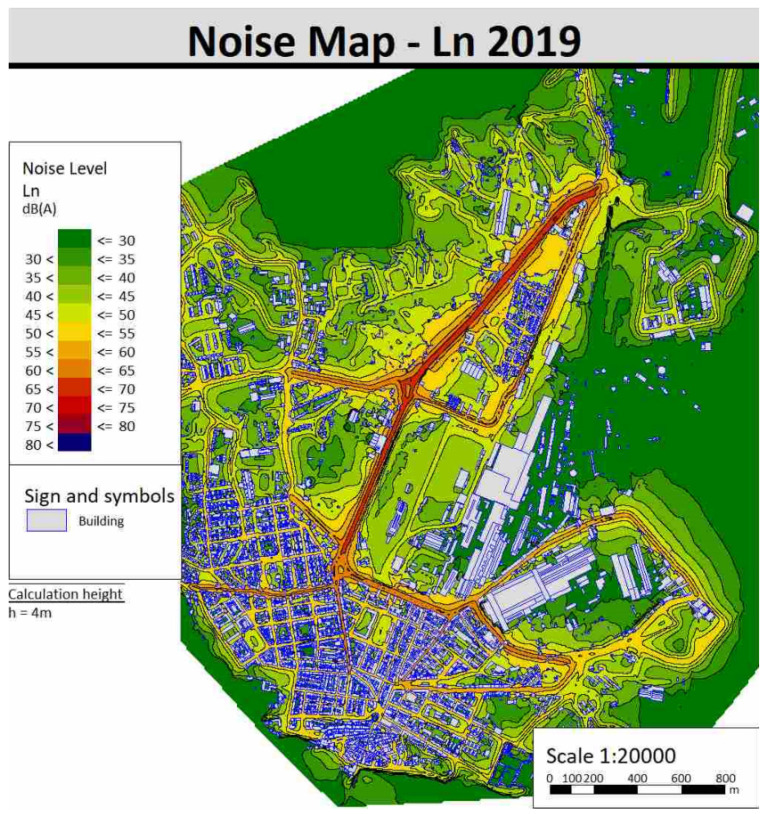
Noise maps of Piombino with L_n_ indicator for 2019.

The acoustic maps of the area were then calculated following the method described in Section 3.4. Figure 13 shows the acoustic mapping according to the L_den_ indicator and Figure 14 shows the one according to the L_den_ indicator for the year 2019. In order to not burden the discussion, the acoustic maps of 2019 carried out with the other indicators (L_d_, L_e_, L_t_) and all that of 2021 are reported in the Supplementary Materials, Figures S1–S8.

**Figure 15:**
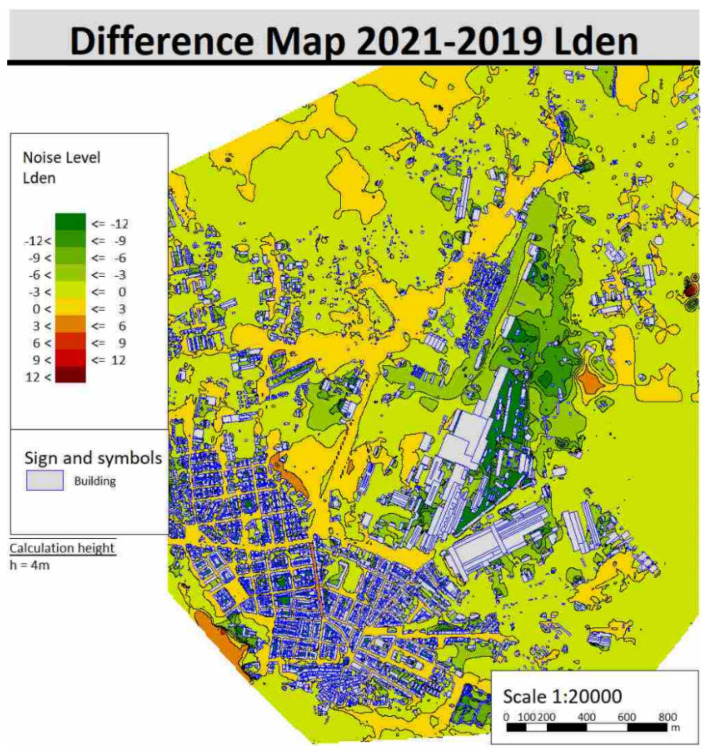
Difference maps of noise for 2021–2019 with L_den_ indicator.

**Figure 16:**
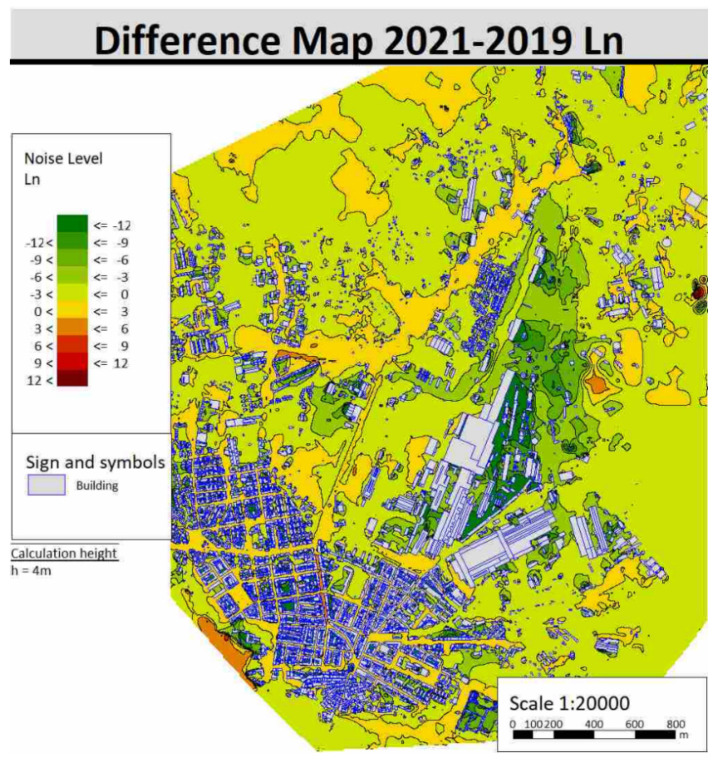
Difference maps of noise for 2021–2019 with L_n_ indicator.

Maps of the noise differences between the year 2019 and the year 2021 were also calculated to show the difference in noise emitted in the area. Differences of L_den_ and L_n_ indicators are shown respectively in Figure 15 and Figure 16 by way of example.

It can be easily inferred which areas show improvements in the acoustic climate and which, on the other hand, have worsened after the activation of the ITS.

However, the efficacy of an intervention should not only be evaluated from an energetic point of view—i.e., the noise emitted into the environment—as it is more important to relate it to the changes in the citizens’ exposure to noise.

**Figure 17:**
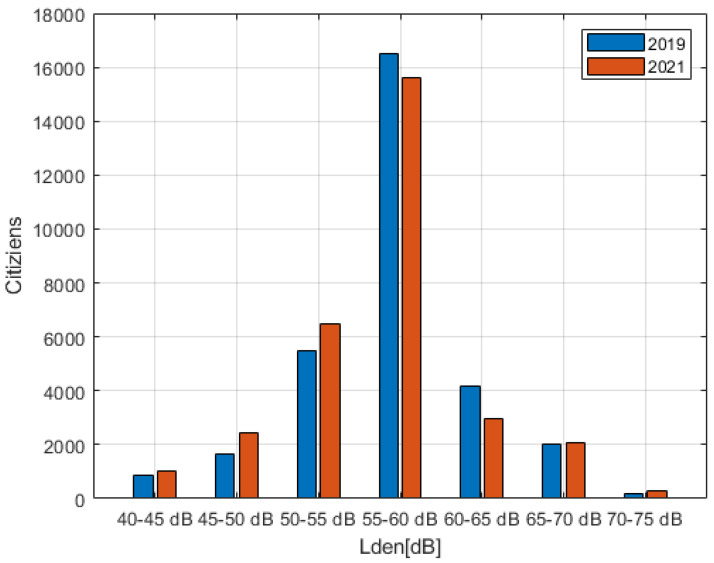
Population exposed to L_den_ exposure categories for both 2019 and 2021.

**Figure 18:**
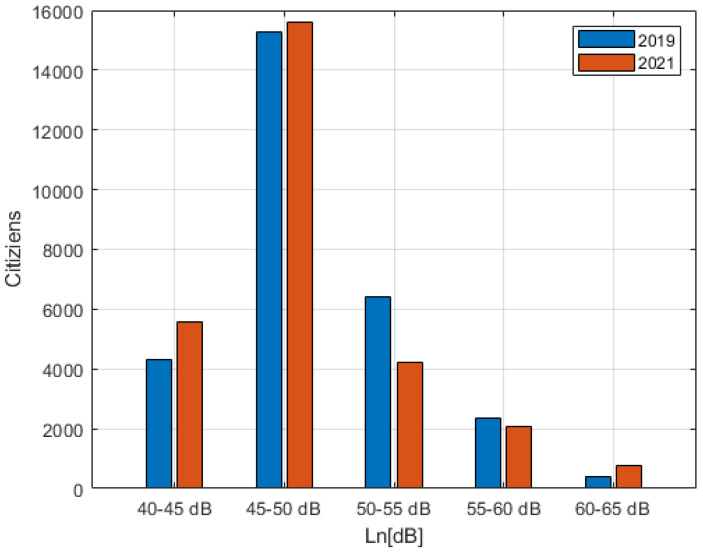
Population exposed to L_n_ exposure categories for both 2019 and 2021.

The calculation of the noise levels of the buildings was then carried out, associating each inhabitant with the maximum level on the façade of its building following the methodology described in the END [10]. The resulting histograms of citizens exposed to the different noise classes is shown in Figure 17 for L_den_, and in Figure 18 for L_n_. 

## 5. Discussion

From the results of Figure 17 and Figure 18, the number of people exposed to the highest levels (70–75 dB (A) for L_den_ and 60–65 dB (A) for L_n_) is increased, while those exposed to medium levels (55–65 dB (A) for L_den_ and 50–60 dB (A) for L_n_) has decreased in favor of an increase in those exposed to lower levels (<55 dB (A) for L_den_ and <50 dB (A) for L_n_).

However, the number of people within each class of exposure is very uneven, and simply comparing the population histograms would lead to neglecting the overall tolerability that the population has of noise. Therefore, to correctly interpret the results, the authors decided to use the group noise indicators first introduced in 2010 by Jabben et al. [64]: the G_den_ and the G_night_. They evaluate the average energy to which the population is exposed, respectively according to L_den_ and L_n_. Their original use was to compare zones of the same city, while in 2013 Licitra and Ascari [65] proposed a revised version more focused on comparing results between European cities. The revised version of the indicators reported in Equation (1) multiply the number of inhabitants by noise energy, with the introduction of a weighting factor on the total inhabitants (N_tot_). This correction helped in identifying the worst-polluted cities and not larger ones as the original indicators did. n_i_ is the population exposed to the i-th class of exposure and L_den_i_ is the representative value of i-th class of exposure. The results are shown in Table 1.

(1) $$G_{\mathrm{den}}=10\cdot \log_{10}\left(\frac{1}{N_{\mathrm{tot}}}\sum_{i}n_{i}\cdot 10^{0.1\cdot L_{\mathrm{den},i}}\right),\;G_{\mathrm{night}}=10\cdot \log_{10}\left(\frac{1}{N_{\mathrm{tot}}}\sum_{i}n_{i}\cdot 10^{0.1\cdot L_{\mathrm{night},i}}\right)$$

The levels of G_den_ and G_night_ in Piombino are well below the national average both in Italy (63.0 dB (A)) and in Europe (63.3 dB (A)) calculated by Licitra and Ascari [65] over all the European cities with more than 100,000 residents, as required by the END [10]. In this study, the noise is only given by the roads and does not include the other main sources of noise (railways, airports, industries) which have instead been included in [52] for the other cities. However, Piombino has no airport, while railways and industries should not represent a significant addition to the overall noise. The reason why the G_den_ in Piombino is lower is probably to be found in the different type of city, smaller than that for which acoustic mapping is mandatory. Therefore, the values provided in [61] can only be used as a reference.

Both G_den_ and G_night_ in Piombino slightly increased between 2019 and 2021, after the inclusion of the ITS. This is a sign that the average energy to which citizens are exposed has also increased.

A final test to verify the efficacy of the ITS as sound mitigation was to verify the health effects due to exposure to noise before and after installation, evaluated through the annoyance and sleep disturbance. As proven by the World Health Organization in “Environmental noise guidelines for the European Region” [79] and from a vast amount of research, these two effects are the most common ones and have a well-defined dose–effect relationship.

The total number of highly annoyed citizens was calculated by applying the curve of Guski et al. [80] shown in Equation (2), for the association between exposure to road traffic noise (L_den_) and the percentage of highly annoyed people (%HA) subjected to L_den_ exposure data.

(2) $$\mathrm{Road}\;\%\mathrm{HA}=78.9270-3.1162\cdot L_{\mathrm{den}}+0.0342\cdot L_{\mathrm{den}}{}^{2}$$

Similarly, the total number of citizens affected by sleep disturbance was calculated using the combined relation of Basner and McGuire [2] on the probability of being highly sleep-disturbed (%HSD) by road traffic noise (Equation (3)) applied to L_n_ exposure data. The results are reported in Table 2.

(3) $$\mathrm{Road}\;\%\mathrm{HSD}=19.4312-0.9336\cdot L_{n}+0.0126\cdot L_{n}{}^{2}$$

The results in Table 2 show how the application of ITS has led to a slight improvement in the health of the population, intended as a reduction in the number of highly annoyed or sleep-disturbed citizens, although the average energy associated with the population has increased due to the increase in traffic flow. The reduction in the number of highly annoyed and sleep-disturbed citizens between 2019 and 2021 is equal to 1.8% and 4.3%.

In summary, the installation of ITS has led to the modification of traffic flows by increasing that in the main access road to the port. Not being specifically designed for noise abatement, this intervention led to an increase in noise exposure of citizens who were already exposed to significant levels of road noise (70–75 dB (A) for L_den_ and 60–65 dB (A) for L_n_). On the other hand, traffic has decreased on the minor roads, which are the ones that run through the most-populated areas and for which the acoustic quality has improved. Therefore, although the average energy associated with citizens (G_den_) has increased, the possible effects on health have slightly decreased. This small result does not justify the use of an ITS as a stand-alone noise mitigation.

The conveyance of traffic along a few major roads has increased the exposure of citizens who already lived in those highly exposed areas, but it can represent an excellent opportunity for further targeted mitigation action. The advantages of installing the ITS could manifest with an eventual new laying of the road surface with low-emission asphalts. In this case, the effect of the redistribution of traffic would reduce the need for pavement replacement while making the acoustic improvement of the entire area much more effective.

The effect of a combined mitigation action between ITS and careful management of new laying of pavements is therefore being evaluated by repeating the previous analyses with the hypothesis of a Zeer Open Asfalt Beton (ZOAB) double-layer pavement on only three roads (Viale della Repubblica, Viale della Resistenza, and Viale Matteotti) for a total length of 1750 m. These roads are those with the highest population among those that suffered from an increase in traffic after the installation of the ITS. A new acoustic modeling was therefore carried out to assign the exposure level to the inhabitants, both in the 2019 and 2021 conditions. Table 3 shows the results of the analyses performed with the low-emission asphalts in both the scenarios with the simultaneous use of ITS (2021) and those without ITS (2019).

The efficacy of each noise mitigation—i.e., asphalts alone, ITS alone, and the combination of both—is reported in Table 4, where the differences of G_den_ and G_night_ and percentage differences of highly annoyed and sleep-disturbed citizens are reported. The percentages obtained by comparing the results of Table 1, Table 2 and Table 3. For “Asphalts alone” the comparison is meant for both scenarios of 2019 with and without the low-noise pavements over the three roads. For “ITS alone” the comparison is meant for scenario 2019 and scenario 2021 both without asphalts. For “Asphalts + ITS” the comparison is meant for scenario 2019 without asphalts with scenario 2021 with asphalts.

## 6. Conclusions

A road traffic monitoring video measurement system (VMS) has been developed and applied for acoustic monitoring. Although its main task is traffic flow and speed measurement to be used as input to noise mappings, the system can be used also for statistical pass-by (SPB) or controlled pass-by (CPB) measurements. The low-cost video recording system (VRS) is based on a single-board computer equipped with an infrared camera sensor and can be used outdoor for long-term acquisition because it is not affected by atmospheric agents. The video analysis system (VAS) includes a trained deep learning YOLOv2 object detection model to detect and classify vehicles in agreement with the categories defined in the CNOSSOS-EU noise assessment model [15].

The VMS has been evaluated over a dataset gathered in several measurement campaigns. It has proven to be reliable by showing good performances with a mean average precision (mAP) equal to 92%. A more specific validation, based on comparisons with other measurement methods present in the literature, will be the subject of future investigations by the authors.

Given its small size, the VRS can be mounted together with sound level meters on a traditional monitoring station placed at roadside position. For collecting the input data necessary for an acoustic map, the quality of data acquired and the number of acquisition points on the territory is important for the overall quality of the outputs of the acoustic model. In this sense, then, the low-cost sensor approach makes the installation of more monitoring stations in urban areas feasible, if compared to other traditional and more expensive acquisition methodologies.

The VAS could be easily updated to be integrated in existing Intelligent Transportation Systems (ITS) for traffic control in a wider context of a traffic-integrated management system which in the future could achieve near real-time updated road noise maps, which would improve the action plans phase. Moreover, noise maps are not only the tool on which action plans are based, but they also represent the best communication tool with citizens. A dynamic and updated map, similar to what recently performed in Dynamap Life Project [81], will better guide the reduction in citizens’ exposure to noise and would allow people to access and monitor the current situation online. This would increase awareness and attention to the issue of noise.

The VMS has been used to acquire traffic data in the city of Piombino, where for the INTERREG Maritime L.I.S.T. PORT Project, an ITS for the management of traffic flows was installed. With the traffic flow and speed data acquired with the developed VMS, acoustic maps of the area were carried out before and after the installation of the ITS. The maps have been validated through short- and long-term noise measurements.

The case study was used to evaluate the effectiveness of the ITS system as a method of acoustic mitigation. In order to do so, the traffic measured in 2019 and 2021 has been normalized to the year 2019, due to the significant difference in flows between the two years entering the city and probably due to the pandemic that encouraged local tourism. The evaluation of the effectiveness was performed by comparing the exposure of citizens to noise, calculating the G_den_ and G_night_ indicators and the number of highly disturbed citizens or with sleep disturbance in ante- and post-operam conditions. The two health effects were estimated using the well-known dose–effect curves in literature.

It has been observed that the inclusion of ITS acted as a focus of traffic in certain roads, corresponding to those already with greater traffic. This resulted in an increase in exposure to citizens who were already exposed to significant levels of road noise (70–75 dB (A) for L_den_ and 60–65 dB (A) for L_n_), but at the same time reduced exposure of those who were exposed to medium noise levels (55–65 dB (A) for L_den_ and 50–60 dB (A) for L_n_) shifting them to lower exposure classes (<55 dB (A) for L_den_ and <50 dB (A) for L_n_). The average energy associated with citizens (G_den_) was increased, but the possible health effects slightly decreased.

Those obtained are modest mitigation results that do not suggest ITS as noise mitigation solution, confirming that it was not designed for this function. However, the effect of conveying traffic to only some roads led the authors to think of simulating the combined effect of ITS with interventions specifically designed to mitigate noise, such as the introduction of low-noise pavements. The combined action of ITS and the laying of asphalts on only three roads, for a total of 1750 m of asphalt, resulted in a significant increase in the mitigation effect that the laying of those asphalts alone would have had without the reorganization of the traffic brought by the ITS. Although this part of the study was carried out as a first test performed in a small city, the rate of improvement brought about by the two actions combined is significant and would improve the quality of life of a greater number of citizens if applied in more densely inhabited cities. Furthermore, the design of the ITS could also be more oriented towards acoustics—i.e., moving traffic towards roads with higher speed limits—where the effectiveness of low-noise pavements is greater.

In summary, the ITS could be tuned according to noise criteria and could represent a dynamic solution for managing traffic in both mapping and action plan phases. This, in addition to enhancing the mitigation effects that would occur with individual interventions, would also represent an economic saving for the administrations in the gathering of input data for noise mapping and for the optimization of the mitigation effects while minimizing the length of intervention.

## Figures and Tables

**Table 1:** G_den_ and G_night_ for Piombino in 2019 and 2021.

Year	G_den_ (dB (A))	G_night_ (dB (A))
2019	59.88	51.20
2021	60.09	51.30

**Table 2:** Total citizens of Piombino highly annoyed and sleep-disturbed in 2019 and 2021.

Year	Highly Annoyed Citizens	Sleep-Disturbed Citizens
2019	3610	979
2021	3545	938

**Table 3:** G_den_ and G_nigh_, total citizens of Piombino highly annoyed and sleep-disturbed in 2019 and 2021 with double-layer ZOAB pavements on three roads.

Year	G_den_ (dB (A))	G_night_ (dB (A))	Highly Annoyed Citizens	Sleep-Disturbed Citizens
2019	59.78	51.09	3597	975
2021	59.11	50.38	3494	929

**Table 4:** Efficacy of the laying of three low-noise pavements, or installation of the ITS or their combination evaluated trough the difference in G_den_ and G_nigh_, or the percentage differences of total citizens of Piombino highly annoyed and sleep-disturbed.

Noise Mitigation	G_den_ (dB (A))	G_night_ (dB (A))	Highly Annoyed Citizens	Sleep-Disturbed Citizens
Asphalts alone	−0.67	−0.70	−2.8%	−4.7%
ITS alone	+0.19	+0.08	−1.8%	−4.3%
Asphalts + ITS	−0.85	−0.98	−4.3%	−7.9%

## Data Availability

Not applicable.

## Supplementary Materials

The following supporting information can be downloaded at: https://www.mdpi.com/article/10.3390/s22051929/s1, Figure S1: Noise maps of Piombino with Ld indicator for 2019; Figure S2: Noise maps of Piombino with Le indicator for 2019; Figure S3: Noise maps of Piombino with Lden indicator for 2021; Figure S4: Noise maps of Piombino with Ld indicator for 2021; Figure S5: Noise maps of Piombino with Le indicator for 2021; Figure S6: Noise maps of Piombino with Ln indicator for 2021; Figure S7: Difference maps of noise for 2021–2019 with Ld indicator; Figure S8: Difference maps of noise for 2021–2019 with Le indicator.
