# Supplementary material for: Traffic Flow Detection Using Camera Images and Machine Learning Methods in ITS for Noise Map and Action Plan Optimization
Source: Sensors (Basel). 2022 Mar 1;22(5):1929. doi: 10.3390/s22051929 (PMC8914976; doi:10.3390/s22051929)
Supplement: Supplementary file 1 [file sensors-22-01929-s001.zip › sensors-1587298-supplementary.pdf]

Supplementary Materials

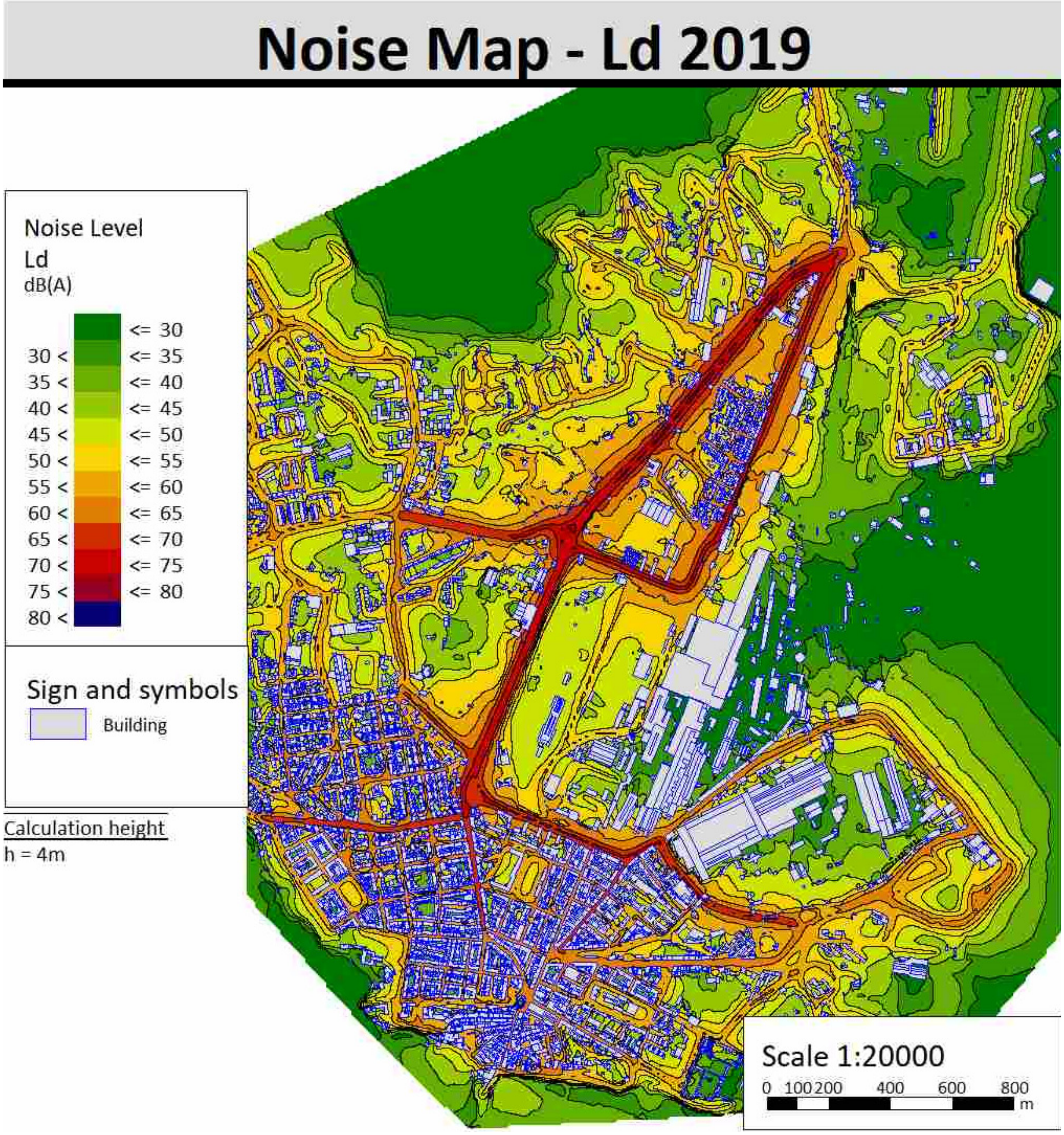

Figure S1. Noise maps of Piombino with L_d indicator for 2019.

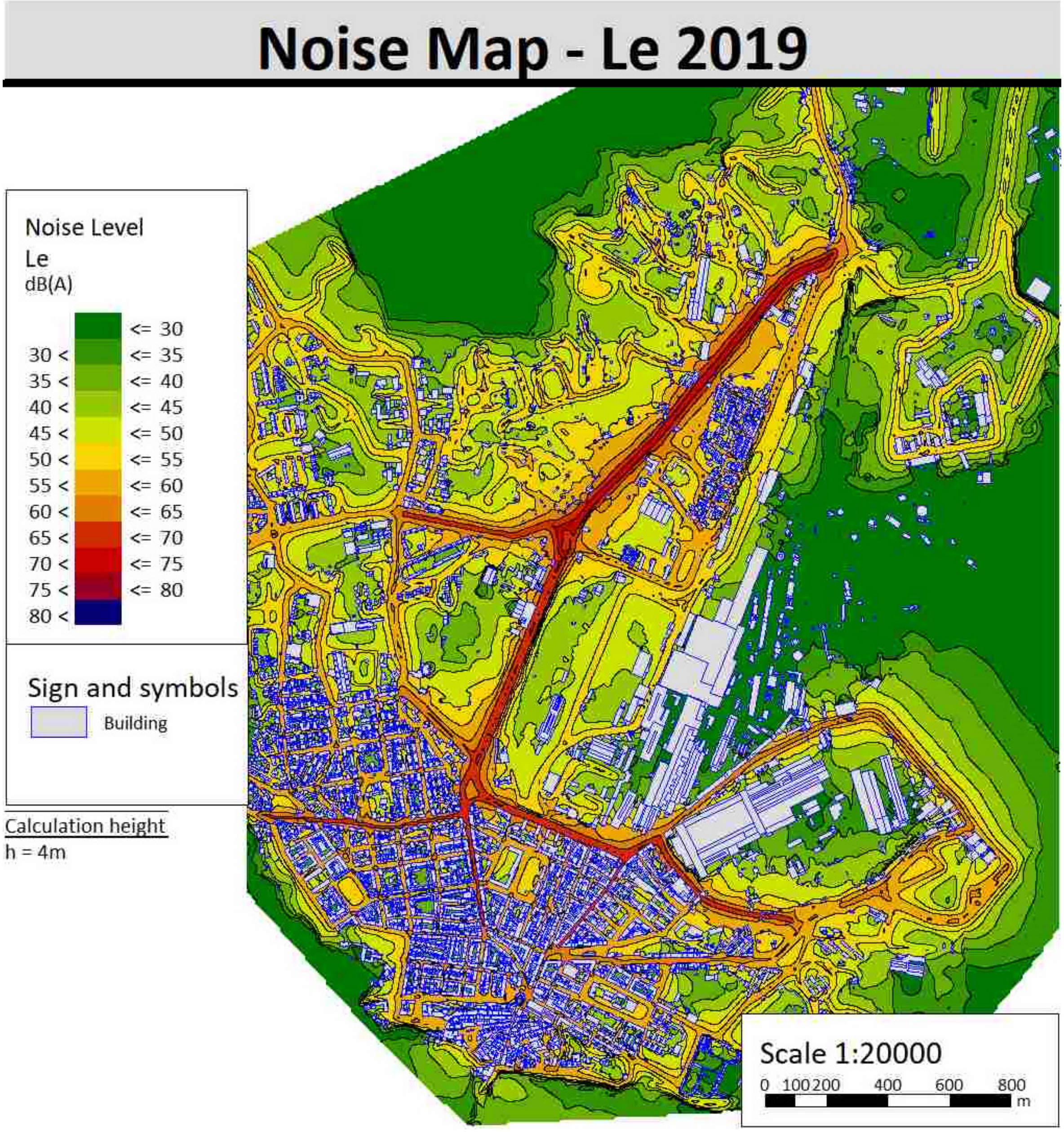

Figure S2. Noise maps of Piombino with L_e indicator for 2019.

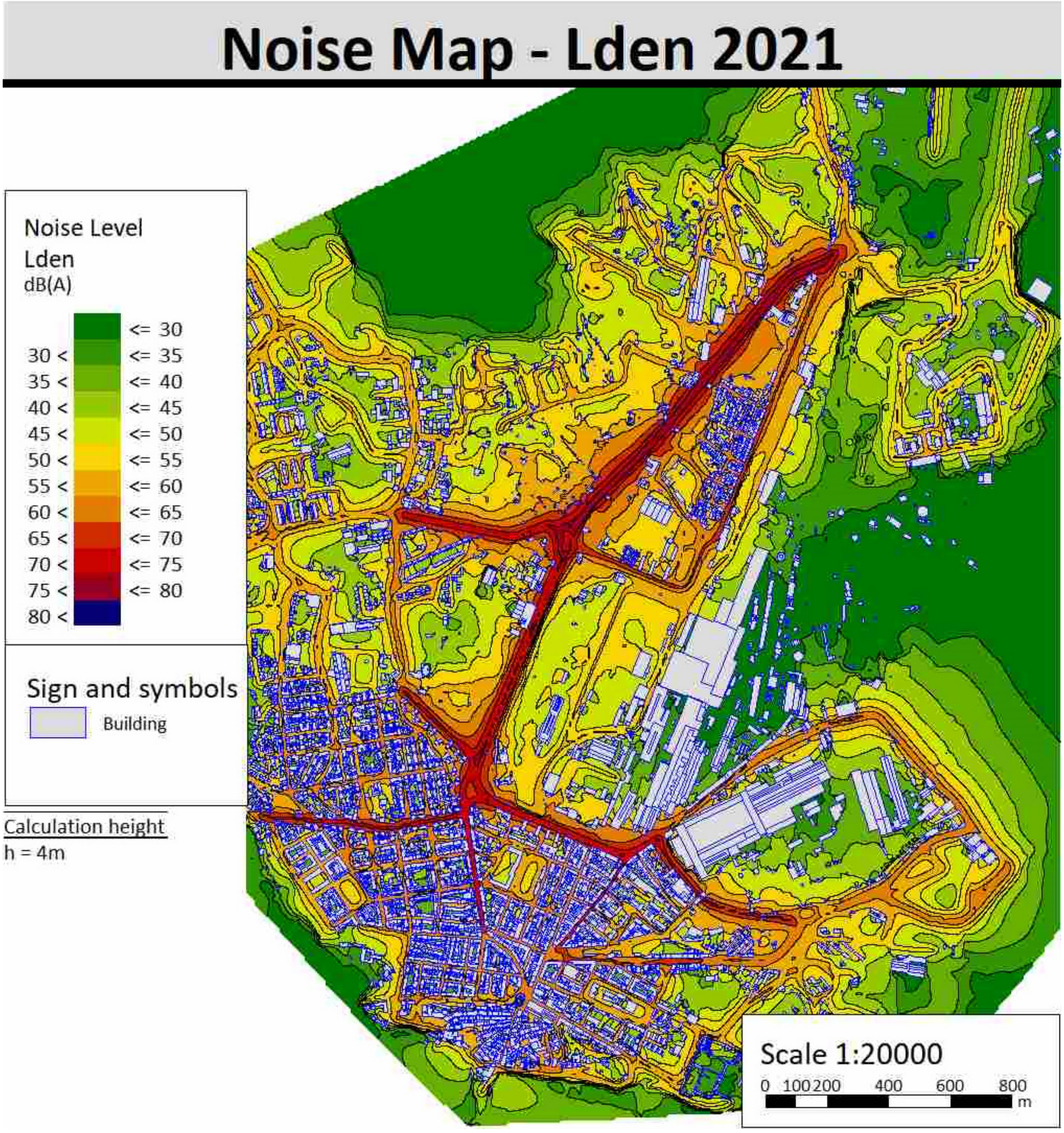

Figure S3. Noise maps of Piombino with L_{den} indicator for 2021.

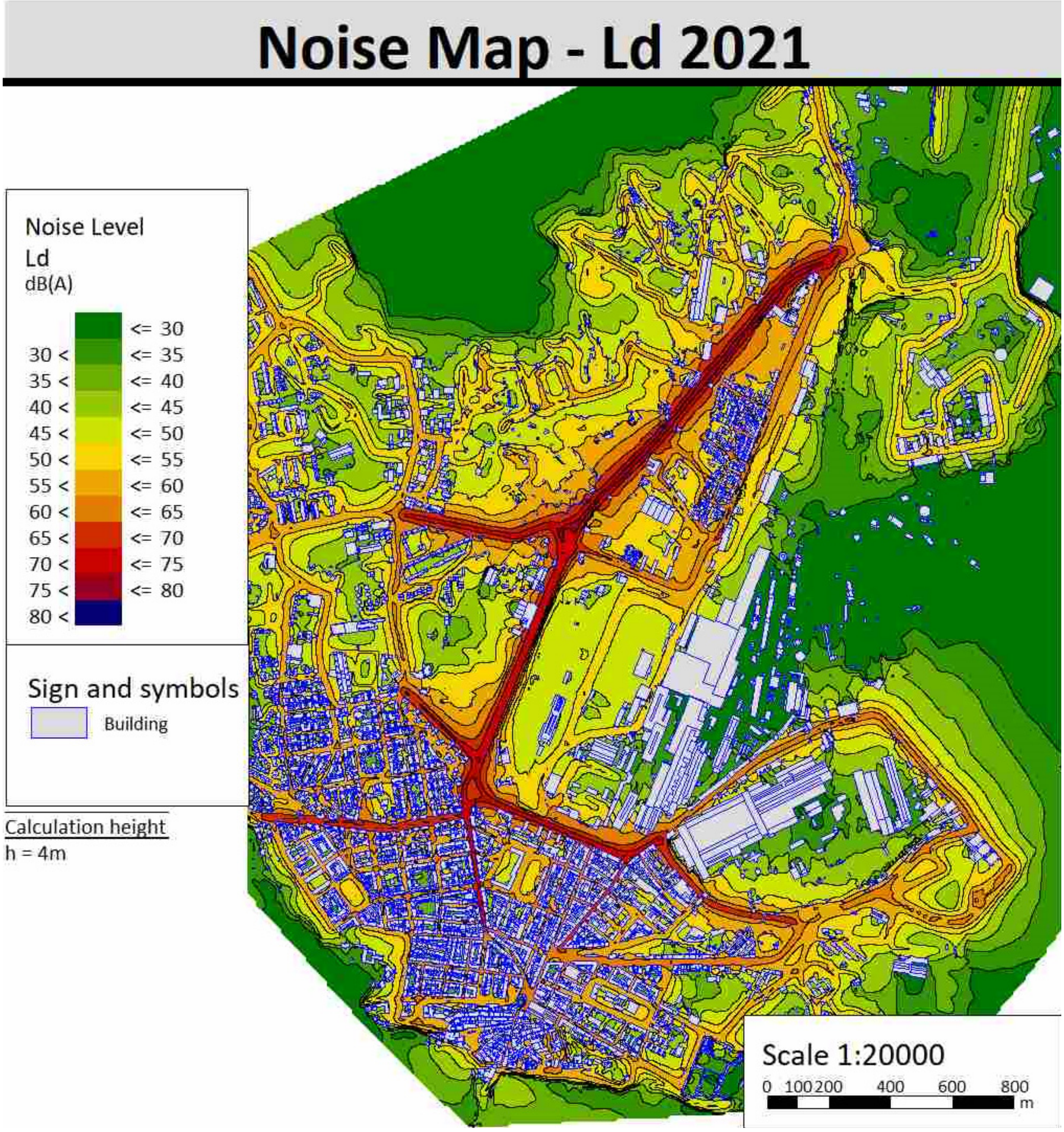

Figure S4. Noise maps of Piombino with L_d indicator for 2021.

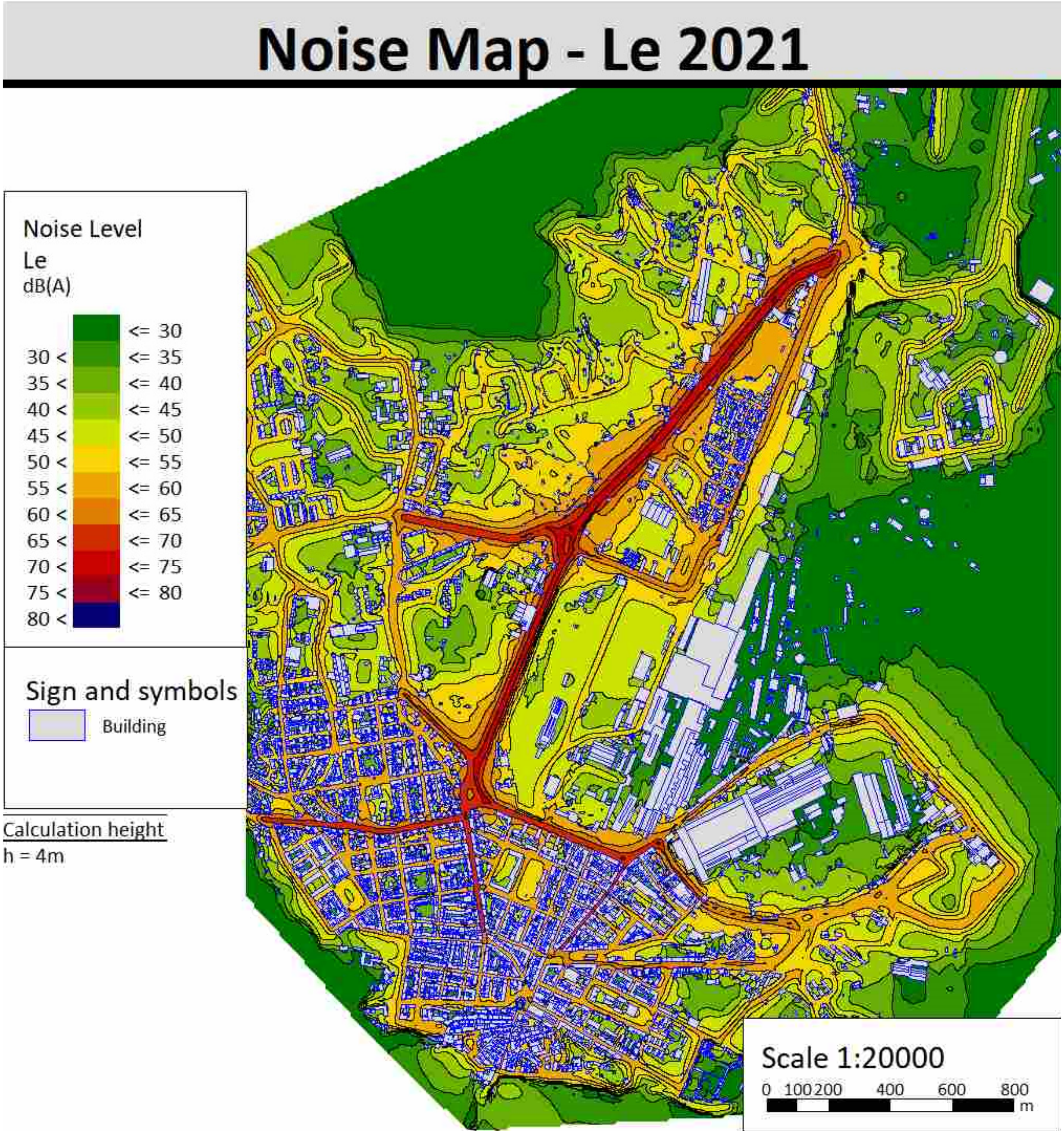

Figure S5. Noise maps of Piombino with L_e indicator for 2021.

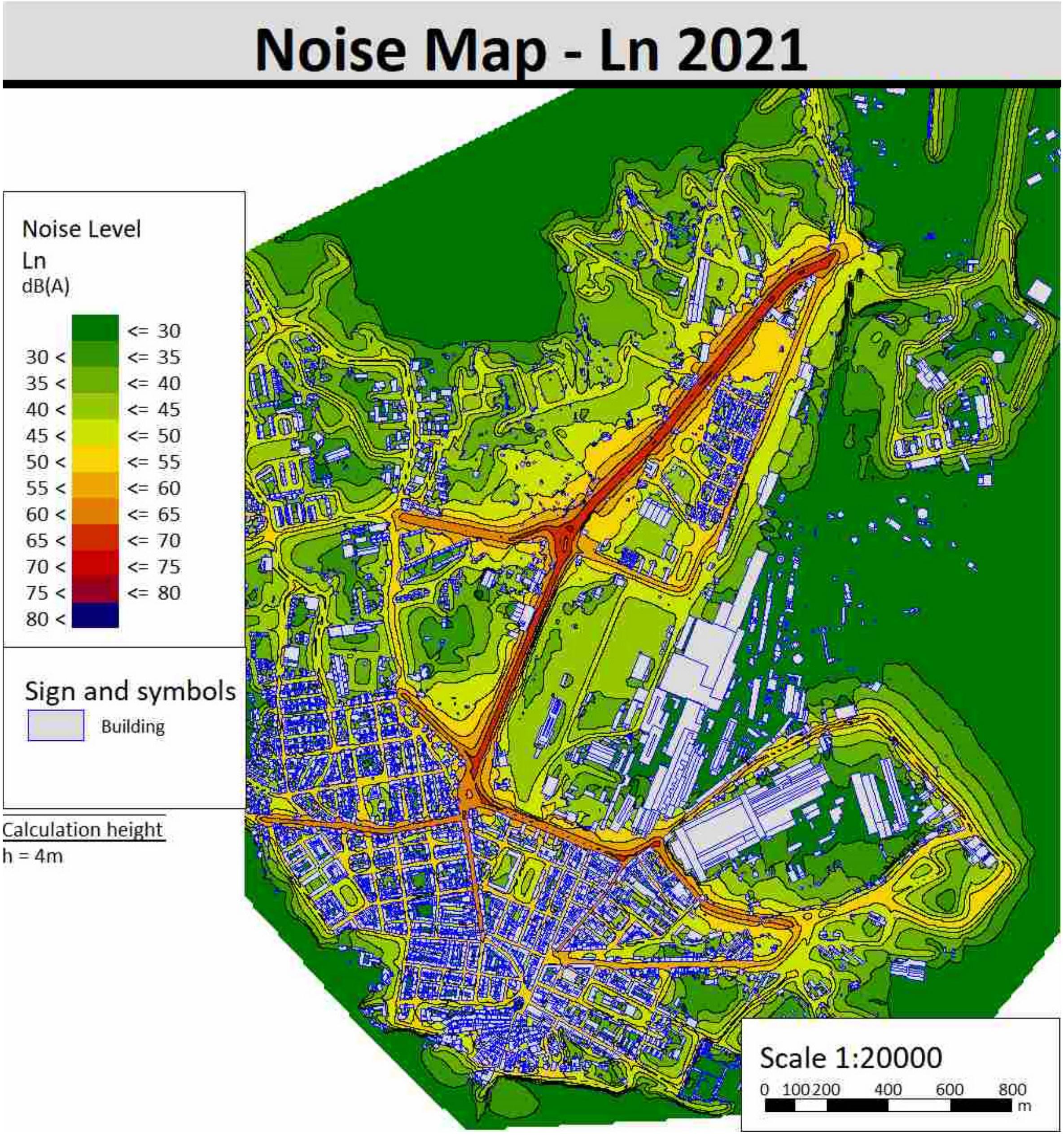

Figure S6. Noise maps of Piombino with L_n indicator for 2021.

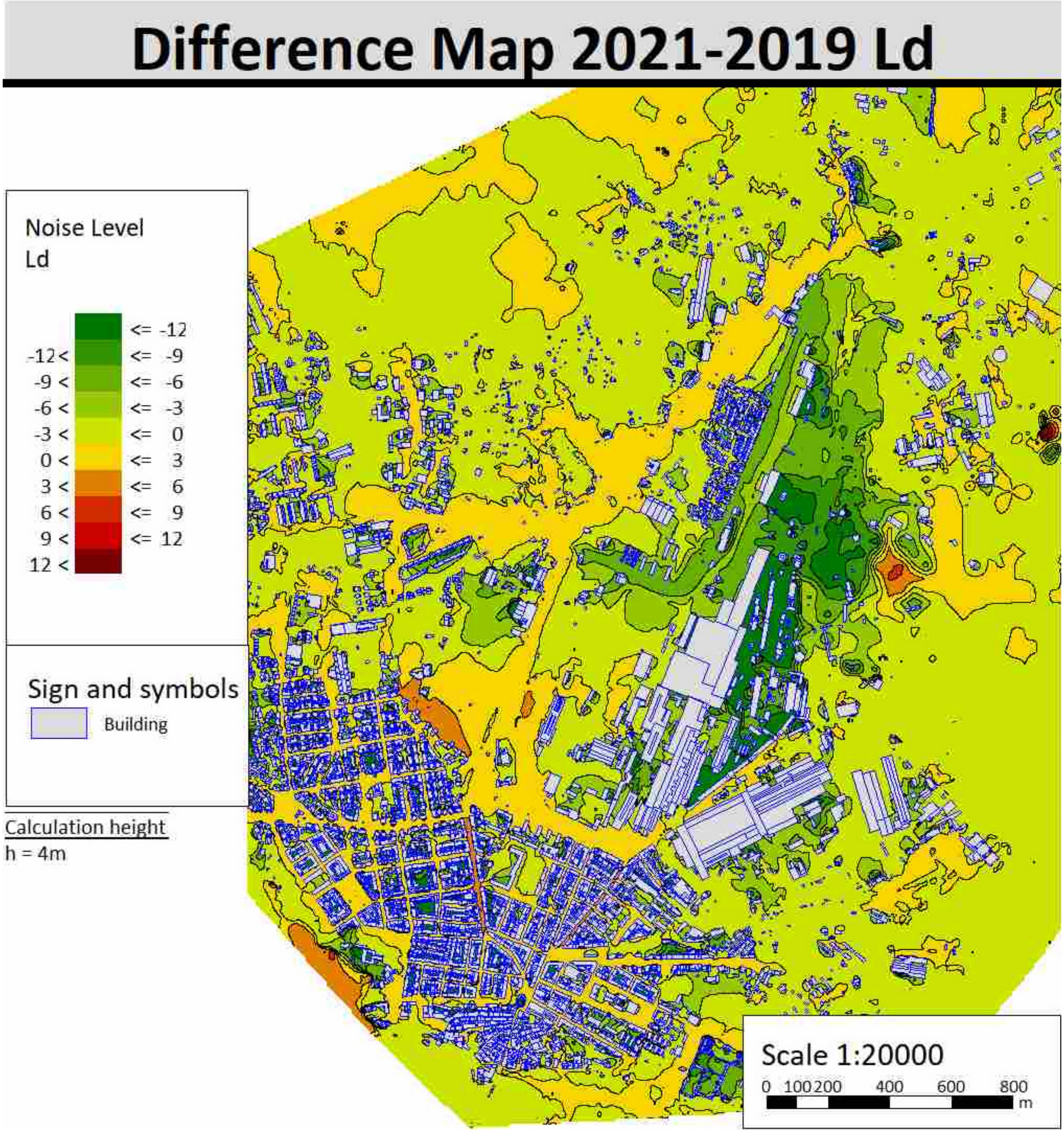

Figure S7. Difference maps of noise for 2021–2019 with L_d indicator.

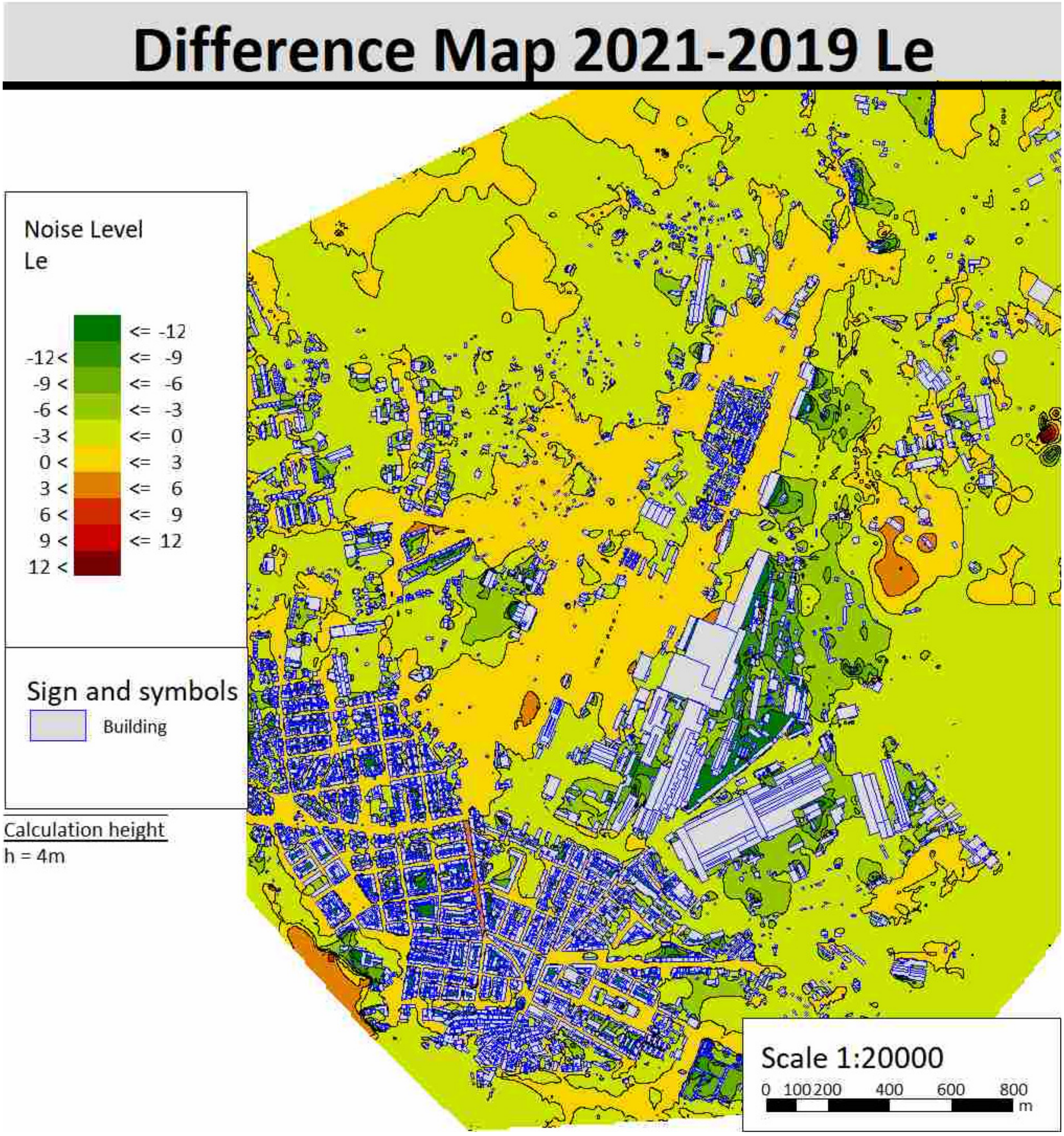

Figure S8. Difference maps of noise for 2021–2019 with L_e indicator.
